# Proprietary Cationic Lipo-Oligopeptides Inhibit Herpes Simplex Virus-1 Infection

**DOI:** 10.3390/microorganisms14102182

**Published:** 2026-09-29

**Authors:** Othreniel A. Forte, Joseph A. Ayariga, Rajnish Sahu, Aguy C. N. Sipowe, Donald R. Owen, Vida A. Dennis

**Affiliations:** 1 Ph.D. Program in Microbiology, Department of Biological Sciences, Alabama State University, Montgomery, AL 36104, USA; oforte3773@alasu.edu (O.A.F.); jayariga@alasu.edu (J.A.A.); anguiakamsipowe0055@myasu.alasu.edu (A.C.N.S.); 2 Center for NanoBiotechnology Research, Department of Biological Sciences, Alabama State University, Montgomery, AL 36104, USA; rsahu@alasu.edu; 3 Owen Biosciences Inc., Baton Rouge, LA 70809, USA; drowen@owenbiosci.com

**Keywords:** antimicrobial peptides, herpes simplex virus type 1, glycoproteins, ADMET, molecular docking, ligand, viral plaque assay, cytotoxicity

## Abstract

The use of antimicrobial peptides (AMPs) to combat herpes simplex virus type 1 (HSV-1) infection has surged amidst the rise of HSV-1 drug-resistant strains. AMPs offer an unconventional approach to combat HSV-1 infection by exerting their activity on multiple phases of the viral life cycle, while exhibiting low rates of resistance emergence. In this study, an integrated in silico and in vitro approach was used to evaluate six proprietary cationic lipo-oligopeptides (CLOPs) (P226, P359, P577, P581, OB1105, and OB1111) as potential anti-HSV-1 agents. Computational tools were used to predict various physiochemical and pharmacokinetic parameters of CLOPs and to assess their molecular docking and binding affinities for HSV-1 glycoproteins. Our findings showed that CLOPs conform to the Pfizer’s rule, predicting a low risk of toxicity. All CLOPs were predicted to exhibit antiviral activity against HSV-1 and interact with major HSV-1 glycoproteins, with energies ranging from −7.5 to −3.2 Kcal/mol. Next, in vitro cytotoxicity along with viral plaque reduction and attachment assays were performed in eukaryotic cells. Our results confirmed that CLOPs were non-cytotoxic and inhibited HSV-1 plaque formation and viral titers in eukaryotic cells. Notably, the plaques were visually smaller in treated cells than those in the HSV-1 control, suggesting a treatment effect on plaque morphology. Among the tested CLOPs, OB1111 emerged as the best anti-HSV-1 candidate, consistently demonstrating strong antiviral activity across both computational and experimental platforms. OB1111 also exerted a significant antiviral effect at the earliest stage of infection in cells. Collectively, these findings identify OB1111 as a strong candidate for further evaluations of its antiviral effects and mechanisms against HSV-1.

## 1. Introduction

Herpes simplex virus type 1 (HSV-1) infection is highly prevalent worldwide, affecting approximately 67% of the global population under 50 years of age in 2016 [1,2]. HSV-1 is an enveloped, double-stranded DNA virus belonging to the Alphaherpesvirinae subfamily that can severely undermine human health by causing a spectrum of neurological diseases [3,4]. Its genome is ~152 kb long and comprises an envelope, a proteinaceous tegument, and a nucleocapsid that protects the viral genome. HSV-1 primarily infects mucosal epithelial cells and establishes lytic infection; after which, the progeny virions travel along axons to infect sensory neurons in the trigeminal ganglia, thereby establishing persistent latent infections [5]. Acyclovir (ACV) and its derivatives can effectively manage HSV-1 infection by reducing the rate of recurrent episodes, lesion formation, and the duration and severity. However, the reliance of these drugs on active viral replication limits their efficacy to lytic infections. To exacerbate this, mutations in viral thymidine kinase and DNA polymerase genes result in drug-resistant HSV-1 strains, which are commonly seen in immunocompromised hosts [4,6,7]. Hence, there is an urgent need for alternative treatments to control HSV-1 infections.

Drug development efforts to combat HSV-1 have primarily focused on lytic infection, with specific priorities given to the initial infection or cell-to-cell spread, which is considered the most promising target for novel antiviral development [8]. HSV-1 has numerous glycoproteins (g) that facilitate its entry into the host cell [9]. Four major glycoproteins drive attachment, entry, and spread, respectively: the receptor binding mediator (gD), the heterodimer fusion regulators (gH/gL), and the viral fusogen (gB) [1]. There is considerable evidence for interactions among glycoproteins, suggesting that they are novel therapeutic targets in HSV-1 drug development. The benefits of targeting HSV-1 glycoproteins extend beyond expanding current antiviral options to combat infection at its initial stages [10]. A few examples include reports by Awasthi and colleagues (2008), who demonstrated that a gD mutant HSV-1 strain reduced cellular viral entry and minimized disease in mice [11]. Similarly, Li et al. (2020) demonstrated that Myricetin, a plant-derived flavonoid, interacts with gD and blocks HSV-1 adsorption and membrane fusion [12]. Another study showed that four peptides designed from HSV-1 gH regions inhibited HSV-1 entry, fusion, and infectivity by membrane insertion and aggregation [13]. Correspondingly, Caesappanin C was found to bind gB and inhibit entry, fusion, replication, and inflammatory response [14]. These studies exemplify the positive effects of limiting HSV-1 infection at the earliest stages by interfering with the function of essential glycoproteins.

Peptide-based therapeutics are emerging alternatives to small molecules that offer several advantages, including high specificity, affordable production rates, and ease of production and optimization [15]. Antimicrobial peptides (AMPs) can destabilize virions, obstruct attachment, entry, replication, and viral egress [16]. Numerous studies have demonstrated their ability to bind to HSV-1 glycoproteins [16,17,18,19,20]. While this is promising, HSV-1 infection remains incurable, and achieving complete viral suppression remains a challenge, warranting continued investigations of alternative peptide candidates.

In this study, we evaluated the potential of six cationic lipo-oligopeptides (CLOPs) as alternative therapeutics against HSV-1 using an integrated in silico and in vitro approach. We hypothesized that CLOPs would be non-cytotoxic, exhibit high affinity for HSV-1 glycoproteins, and effectively inhibit HSV-1 plaque formation in eukaryotic cells. To test our hypothesis, we conducted in silico modeling followed by in vitro cytotoxicity and anti-HSV1 experiments. We present our findings supporting CLOPs as novel, safe, and potentially effective alternative antiviral candidates against HSV-1.

## 2. Materials and Methods

### 2.1. In Silico Peptide Selection and Preparation

The proprietary CLOPs used in this study were provided by Owen Biosciences, Inc. (Baton Rouge, LA, USA). Six CLOPs (P226, P359, P577, P581, OB1105, and OB1111) were selected from a larger group of CLOPs for screening based on their computational antiviral predictions and structural characteristics deemed conducive to binding to enveloped viruses. They are amphipathic and contain highly cationic amino acid residues, particularly Lys and Arg, which favor electrostatic interactions with negatively charged viral membranes, capsids, and proteins, thereby disrupting virions [21]. The presence of the lipid moiety was also considered as an important criterion, as it is known to improve stability, half-life [22], specificity, permeability, and efficacy of AMPs [23]. The molecular structures of these CLOPs were optimized using specific computational tools as described below. The list of tools used in the in silico analysis is presented in Supplementary Table S1.

The selection workflow proceeded from the larger proprietary CLOPs library to computational and structural screening, followed by selection of the six candidates and subsequent experimental testing. Because computational predictions were used during candidate preselection, the tested panel may be enriched for compounds with favorable predicted properties, and these were considered when interpreting the relative performance of the CLOPs.

#### 2.1.1. Lipinski Rule, Pfizer Rule, and Pharmacokinetics

The pharmacokinetic characteristics and drug-likeness score of CLOPs were obtained using SwissADME (https://www.swissadme.ch/, accessed on 19 September 2026) and AMDETlab 3.0 (https://admetlab3.scbdd.com/, accessed on 19 September 2026). These parameters were used as hallmarks to predict the bioavailability and drug-likeness of CLOPs. The Lipinski rule was used to assess the tendencies of CLOPs to conform to conventional drug evaluation guidelines. On the other hand, the Pfizer 3/75 rule was used to evaluate the potential risk of CLOPs-related toxicity. Taken together, the measured parameters included their molecular weight (MW), logP (log of the partition coefficient), number of hydrogen bond donors (nHBD), number of hydrogen bond acceptors (nHBA), and TPSA (topological polar surface area), among others. All physicochemical, drug-likeness, and ADMET calculations were performed on the complete CLOP chemical structures, including the N-terminal lipid moiety and C-terminal amidation; therefore, the reported values correspond to the intact lipo-oligopeptides rather than to the peptide backbone alone.

#### 2.1.2. ADMET (Absorption, Distribution, Metabolism, Excretion, and Toxicity) Analysis

The ADMET properties of CLOPs were predicted using the software tool ADMET Predictor (http://admet.bioai-global.com/predict, accessed on 19 September 2026). Key parameters, including absorption potential, distribution volume, metabolic stability, excretion rates, and toxicity (including hepatotoxicity and cardiotoxicity), were evaluated.

#### 2.1.3. Generation of CLOPs 2D Chemical Structures

The CLOPs under study are proprietary and thus are unavailable in public databases. To easily visualize and interpret the antiviral properties of CLOPs, their amino acid sequences were converted into 2D structures and canonical SMILES using DataWarrior (https://openmolecules.org/name2structure.html, accessed on 19 September 2026) and Open Parser for Systematic IUPAC Nomenclature (OPSIN) (https://www.ebi.ac.uk/opsin, accessed on 19 September 2026) software programs. DataWarrior, (Version 6.2.5 (OpenMolecules)), a data analysis and visualization program, was used to extract hidden knowledge from the CLOPs. For instance, this data filter was used to focus on specific data subsets or to make chemical intelligence work seamlessly with the program. It was used to filter data by various structural motives, view chemical structures from a chemistry-aware perspective, and to predict the molecular properties of CLOPs. DataWarrior supports numerous file types and can merge data from sources or databases. The OPSIN (version 2.8.0) software was used to interpret the International Union of Pure and Applied Chemistry (IUPAC) nomenclature for the structures of various lipopeptides. It converted these structures into three different chemical groups: CML (Chemical Markup Language), InChI (International Chemical Identifier), and SMILES (Simplified Molecular Input Line Entry System). Thus, each CLOP was run through these two programs to generate its 2D structure and canonical SMILES for downstream computational investigations.

#### 2.1.4. Antiviral Peptide Prediction and Validation Workflow

**Figure 1:**
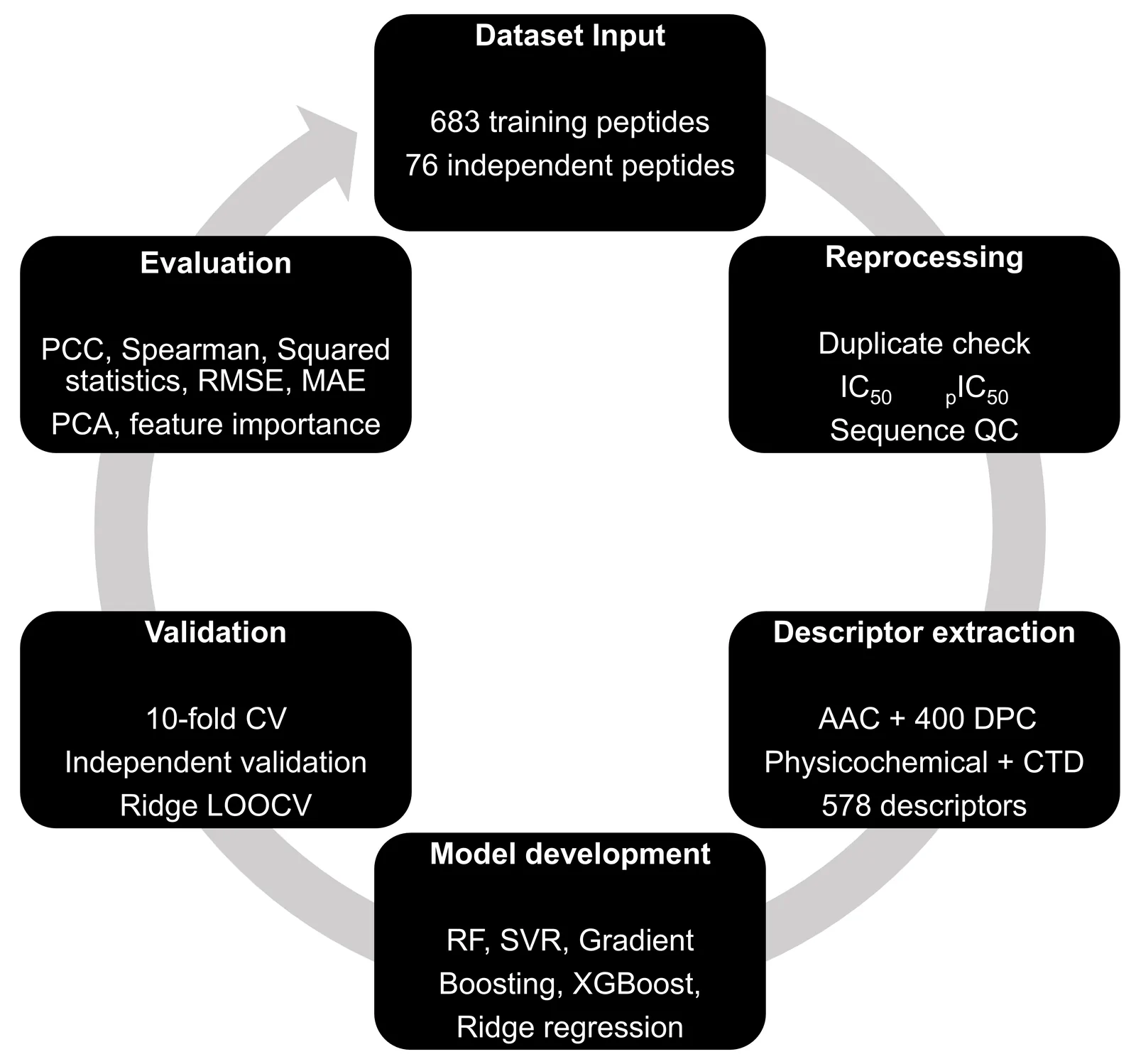
Overall workflow of the antiviral peptide IC_50_ prediction and validation pipeline.

The precision of the machine-learning algorithms (SVR and RF) was assessed in predicting the antiviral activity of AMPs. The workflow, as shown in Figure 1, comprised several steps, including dataset acquisition, in which data were collected and preprocessed to remove duplicate sequences and minimize bias. Experimental IC_50_ values were logarithmically transformed to pIC_50_, which are more suitable for most models. Descriptor extraction aimed to ensure that the peptide sequences were compatible with the models, generating 578 descriptors. Next, the models were developed and trained to understand patterns that distinguish descriptors from the peptide’s antiviral activity. The models’ performance was subsequently validated and evaluated to determine their precision.

#### 2.1.5. Dataset Collection and Preprocessing

The antiviral peptide datasets employed for the machine-learning model development and validation in this study were obtained from the AVP-IC50Pred dataset. This benchmark dataset was originally compiled by Qureshi et al. (2015) [24]. It consists of experimentally characterized antiviral peptides with reported half-maximal inhibitory concentration (IC_50_) values. The model-development dataset contained 683 peptides, while a separate independent external-validation dataset contained 76 peptides. The two datasets were processed separately so that the external-validation peptides were not used for model fitting, feature selection, or hyperparameter optimization. Duplicate sequences were screened within the model-development data, and sequence overlap between the development and external-validation sets was checked before model evaluation. Thus, the development and external-validation sets together comprised 759 peptide records before any within-set duplicate handling. Experimental IC_50_ values (µM) were transformed to pIC_50_ values for modeling because IC_50_ values spanned several orders of magnitude. Basic sequence statistics, amino acid composition, and pIC_50_ distributions are summarized in Supplementary Figure S3. The proprietary CLOPs are 5–6 amino acids long, whereas the training set peptides span 8–38 residues. Therefore, predictions for CLOPs fall outside the training-set peptide-length range and are treated as extrapolative prioritization estimates rather than experimentally validated quantitative IC_50_ values. The formula below was used to convert the IC_50_ to pIC_50_ for all CLOPs.

*pIC*_50_ = 6 − *log*_10_(*IC*_50_ [μM])

#### 2.1.6. Sequence Descriptor Generation

A comprehensive panel of sequence-derived descriptors was generated for each peptide to capture compositional and physicochemical characteristics relevant to antiviral activity. The descriptor set included: amino acid composition (AAC), dipeptide composition (DPC) (comprising 400 descriptors), MW, net charge, pI, aromaticity, GRAVY hydrophobicity index, instability index, aliphatic index, Boman index, sequence entropy, and CTD descriptors. Collectively, these features generated a total of 578 numerical descriptors for each peptide.

#### 2.1.7. Machine-Learning Model Development

Five regression algorithms were evaluated for predicting the antiviral activity. These include Random Forest (RF), Support Vector Regression (SVR), Gradient Boosting Regression, Extreme Gradient Boosting (XGBoost), and Ridge Regression. Following this, the model performance was assessed using three complementary validation strategies: ten-fold cross-validation, independent external validation, and leave-one-out cross-validation (LOOCV). The LOOCV analysis was performed using Ridge regression because exhaustive LOOCV of nonlinear ensemble models would require repeated fitting of hundreds of computationally intensive models.

#### 2.1.8. Model Training Steps

The antiviral potency modeling workflow was implemented as a regression problem because the response variable was continuous IC_50_/pIC_50_. Hyperparameters for the SVR and other regression models were optimized within the training data, and model performance was evaluated using correlation- and error-based metrics appropriate for continuous outcomes. The final validation framework comprised ten-fold cross-validation, an independent external-validation dataset, and LOOCV. Classification metrics such as accuracy, precision, recall, F1-score, ROC, and AUC were therefore not used for the final IC_50_ prediction analysis.

#### 2.1.9. Model Evaluation

Model performance was assessed using Pearson correlation coefficient (PCC), Spearman’s rank correlation coefficient (ρ), coefficient of determination (R^2^), predictive squared correlation coefficient (Q^2^), root mean square error (RMSE), and mean absolute error (MAE). R^2^ and Q^2^ are conceptually distinct metrics and are defined separately in Supplementary Table S3.

#### 2.1.10. Prediction of CLOPs Activity

For quantitative prediction of antiviral activity, experimentally validated antiviral peptides with reported IC_50_ values were used as the modeling dataset. After preprocessing and duplicate removal, sequence-derived descriptors were generated and used to train regression algorithms to predict the pIC_50_/IC_50_. The proprietary CLOPs were encoded using the same descriptor pipeline and submitted to the trained models. SVR, Random Forest regression, XGBoost, Gradient Boosting, and Ridge regression were evaluated, with model performance assessed by PCC, Spearman correlation, squared statistics, RMSE, and MAE. Details of dataset composition, descriptor generation, model development, and validation are provided in Section 2.1.5, Section 2.1.6, Section 2.1.7 and Section 2.1.9. These sequence-derived descriptors represent the amino acid sequences and do not explicitly encode the N-terminal lipid moiety or the C-terminal amidation of CLOPs. Accordingly, CLOPs IC_50_ predictions are interpreted as exploratory prioritization estimates.

#### 2.1.11. Retrieval, Homology Modeling, and Preparation of HSV-1 Glycoprotein Structures

To conduct the in silico modeling analyses, the 3D structures of HSV-1 glycoproteins used for molecular docking were obtained from a combination of experimentally resolved structures deposited in the Protein Data Bank (PDB) and homology modeling approaches. A detailed search of the Protein Data Bank (PDB) was first conducted to identify available crystal or NMR structures of HSV-1 glycoproteins involved in viral attachment, membrane fusion, entry, and immune evasion. This was followed by the identification of experimentally resolved structures for glycoprotein B (gB; PDB ID: 3NW8), glycoprotein D (gD; PDB ID: 2C36), and glycoprotein H (gH; PDB ID: 2LQY). These were downloaded directly from PDB and used as receptor structures for the molecular docking studies. However, some experimentally determined structures were unavailable for several HSV-1 glycoproteins; thus, the amino acid sequences corresponding to glycoproteins gC, gE, gG, gI, gJ, gK, gL, gM, and gN were retrieved from the National Center for Biotechnology Information (NCBI) GenBank database using their respective accession numbers as indicated in Supplementary Table S2. The retrieved sequences were subjected to homology modeling using the Swiss-Model server (https://swissmodel.expasy.org, accessed on 19 September 2026). The 3D protein models were generated using the highest-ranking templates and evaluated using the Global Model Quality Estimation (GMQE) and QMEAN scoring metrics from the Swiss-Model platform (Supplementary Table S4). These highest quality models were selected for downstream molecular docking analyses. Following model selection, both experimentally resolved and homology modeled proteins underwent structural preparation and optimization. Crystallographic water molecules, co-crystallized ligands, ions, and other nonessential heteroatoms were removed using Discovery Studio Visualizer (version v21.1.020298 (Dassault Systèmes BIOVIA)) and PyMOL (version 3.0 (Schrödinger, LLC)). Hydrogen atoms were added, bond orders were verified, and protein structures were visually inspected for structural inconsistencies. Where necessary, structural refinement and energy minimization were performed to improve geometric stability and remove unfavorable steric interactions. The prepared protein structures were subsequently converted into docking-compatible formats, and Kollman charges and polar hydrogens were assigned prior to molecular docking simulations using AutoDock Tools (version 1.5.7).

#### 2.1.12. Molecular Docking and Binding Studies

Molecular docking was used to predict the binding affinities of CLOPs to HSV-1 glycoproteins essential for viral attachment, entry, immune evasion, and spread. The final docking panel comprised gB, gC, gD, gE, gG, gH, gI, gJ, gK, and gL. The modeled 3D structures of the CLOPs were docked to these glycoproteins using PyRx/AutoDock Vina (version 0.8 (Sarkis Dallakyan, The Scripps Research Institute)), with receptor and ligand preparation performed as described above [25,26]. Ligand structures were energy-minimized and converted to PDBQT format, while receptor preparation included addition of polar hydrogens and assignment of appropriate charges. Each receptor grid box was generated in PyRx using the Vina Wizard, and docking was performed with an exhaustiveness of 8. Nine poses were generated for each ligand–receptor pair, and the best-scoring pose was retained for residue interaction analysis in BIOVIA Discovery Studio. Root-mean-square deviation (RMSD) values reported in this study were calculated from the set of docking poses generated for each ligand–receptor pair and therefore describe docking-pose dispersion/consistency. They were not derived from a molecular dynamics trajectory and should not be interpreted as time-resolved structural stability.

### 2.2. In Vitro Validation of CLOPs’ Antiviral Activity

#### 2.2.1. Cell Line and CLOPs

Vero cells (African Green Monkey Kidney cells) were kindly provided by Dr. Francois Villinger (MD Anderson Cancer Center, Bastrop, TX, USA). HEp-2 cells (Human epithelial cells) (ATCC CCL-23) were obtained from the American Type Culture Collection (ATCC, Manassas, VA, USA). Cells were cultured in Eagle’s Minimal Essential Medium (EMEM) (ATCC) supplemented with 10% heat-inactivated Fetal Bovine Serum (FBS), and 1 μg/mL antibiotic and antimycotic (ThermoFisher Scientific, Waltham, MA, USA)). Both cell lines were maintained at 37°C in a humidified incubator with 5% CO_2_. Proprietary CLOPs were provided by Owen Biosciences Inc (see above), and acyclovir (ACV) was purchased from Sigma-Aldrich (St. Louis, MO, USA). CLOPs used in this study were 5–6 amino acids in length, synthesized by Owen Biosciences, Inc., and have a purity grade of >85% as determined by HPLC (unpublished data). Lyophilized CLOPs were dissolved in distilled water, aliquoted, and stored at −80 °C. Aliquots of 10 mg/mL were prepared and stored at −20 °C until use. This stock solution was used to prepare the subsequent working concentrations employed in the study.

#### 2.2.2. Infectivity

HSV-1 strain F (ATCC #VR-733) was obtained from ATCC and propagated in Vero cells monolayers in EMEM supplemented with 2% FBS for 1 h (hour) to establish infection. The cells were shaken periodically to ensure optimal absorption. The infectious inoculum was removed from cells, followed by treatment with CLOPs, which were maintained at 37 °C in a humidified incubator with 5% CO_2_.

#### 2.2.3. Cytotoxicity Studies

The cytotoxicity of CLOPs and ACV to HEp-2 and Vero cells was evaluated by the 3-(4,5-dimethyl-thiazol-2-yl)-2,5-diphenyl-tetrazoliumbromide (MTT) dye reduction assay using an MTT Cell Proliferation Assay kit (ATCC #30-1010K). Cells (5 × 10^4^) were seeded into each well of a 96-well plate in 100 µL of EMEM for 24 h, and incubated at 37 °C in a humidified 5% CO_2_ atmosphere. The culture media were replaced with 100 µL of fresh media containing various concentrations of CLOPs and ACV (0.9 to 250 µg/mL), and incubated for 24 and 48 h. MTT dye solution (10 µL) was added to each well, and incubated for 1.5 and 1 h, respectively, for HEp-2 and Vero cell lines. The detergent reagent (100 µL) was added to each well to dissolve the formazan crystals, followed by incubation overnight at 37 °C in a humidified 5% CO_2_ atmosphere. The absorbance at 570 nm was measured using the Multiskan SkyHigh microplate reader (ThermoFisher Scientific), and cell viability (%) was assessed by comparing the absorbance of CLOPs-treated cells to that of untreated control cells.

#### 2.2.4. Plaque Reduction Assay

The plaque reduction assay was performed using Vero cells, a model cell line for evaluating HSV-1 plaque formation [27]. Vero cells were seeded at a density of 1 × 10^6^ cells/well in 6-well plates for 24 h. The medium was removed, and cells were infected with HSV-1 at a multiplicity of infection (MOI) of 0.001 for 1 h. The inoculum was removed and replaced with 1.2% carboxymethylcellulose (Sigma-Aldrich) alone or containing ACV or CLOPs, according to the experimental design, for 48 h, and the culture was maintained at 37 °C in a humidified incubator with 5% CO_2_. Cells were fixed with 4% paraformaldehyde (ThermoFisher Scientific) and stained with 1% crystal violet (ThermoFisher Scientific) to visualize and enumerate plaques.

#### 2.2.5. Viral Attachment Assay

A viral attachment assay was conducted to evaluate the effect of OB1111 on viral entry. Vero cells were seeded at a density of 1 × 10^6^ cells/well in 6-well plates and cultured for 24 h. Cells, HSV-1, and 2% FBS media were precooled at 4 °C for 30 min, and cells were infected and treated simultaneously with HSV-1 at an MOI of 0.001 and incubated at 4 °C for 1 h. The inoculum was removed, the cultures were replaced with 2% FBS media, and were further incubated at 37 °C in a humidified incubator with 5% CO_2_ for 1 h to permit entry. The media was removed and replaced with 1.2% carboxymethylcellulose, and cultures were maintained at 37 °C for 48 h. Cells were fixed with 4% paraformaldehyde and stained with 1% crystal violet to visualize and enumerate plaques. Plaque inhibition percentages were determined using the formula: Viral inhibition (%) = [1 − (number of plaques) _inhibitor_/(number of plaques) _control_] × 100 [28].

### 2.3. Statistical Analysis

Data from the molecular docking analysis and machine-learning analyses were summarized using the statistical procedures described above. Cell viability data were analyzed using two-way analysis of variance (ANOVA (Graph Pad, Prism 10). HSV-1 plaque-reduction data were analyzed using one-way ANOVA followed by Šidák’s multiple comparison test. Data from the early-stage attachment assay were analyzed using one-way ANOVA, with Bartlett’s test used to assess homogeneity of variances in Prism 10 (GraphPad Software, Inc., San Diego, CA, USA). *p* Values < 0.05 were considered significant.

## 3. Results

### 3.1. CLOPs 2D Chemical Structure and Molecular Features

**Figure 2:**
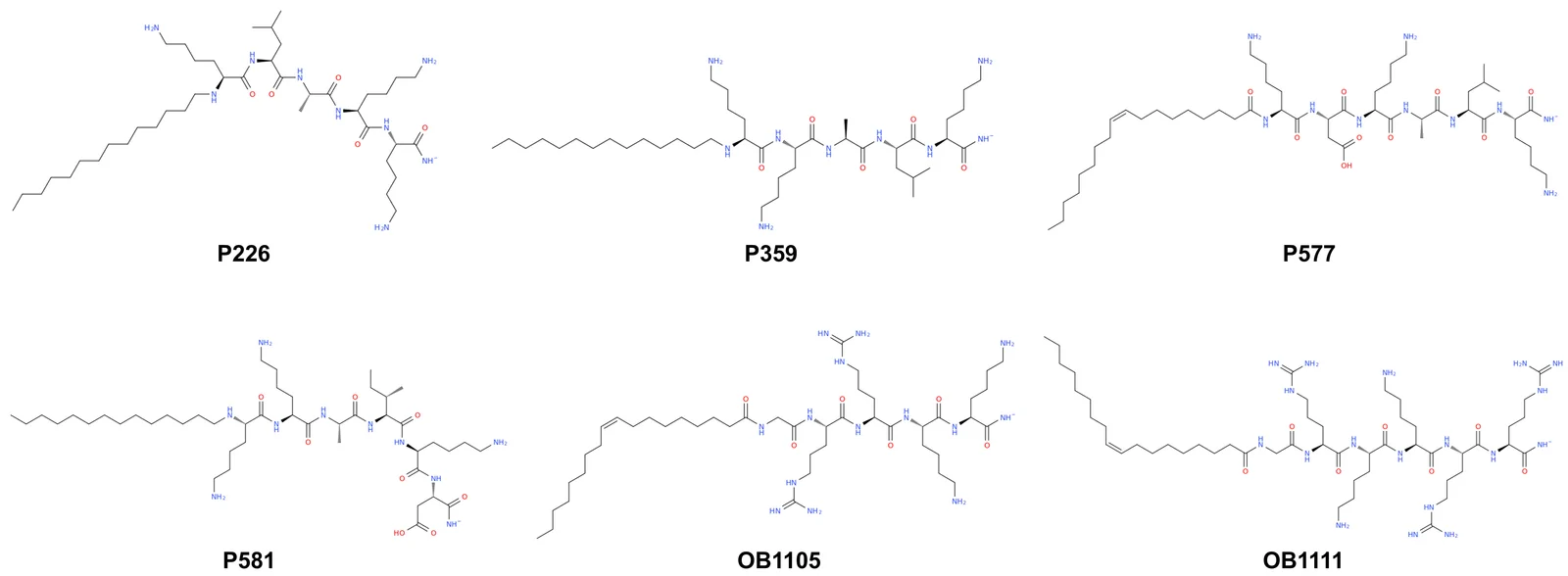
Depiction of the 2D chemical structures of six CLOPs. The amino acid sequences of CLOPs were used to generate their 2D chemical structures using DataWarrior and OPSIN software.

The amino acid sequences of CLOPs were used to generate their 2D chemical structures using the DataWarrior and OPSIN software (Figure 2). All CLOPs used in this study were lipidated to improve cellular uptake, delivery, and stability.

The molecular features of CLOPs, including their composition, net charges, structural features, and modifications, which influence their biological activity, are tabulated in Table 1. CLOPs are positively charged with values ranging from (+3) to (+5), suggesting they can readily bind to negatively charged membranes and cellular surfaces. P226, P359, P577, and P581 are pentapeptides with a net positive charge of (+3), which is mainly attributed to the presence of Lys residues. Likewise, OB1105 is a pentapeptide but differs from its preceding counterparts in charge and amino acid composition. The (+4) net charge results from its Lys/Arg amino acid composition. OB1111 is a slightly more cationic hexapeptide (+5) that is rich in Lys and Arg. Cationic charge directly correlates with biological activity, where a higher charge results in enhanced activity, and vice versa [29]. On this premise, OB1111 and OB1105, with charges of (+5) and (+4), respectively, may have a greater tendency to bind to membranes than the other CLOPs. A defining feature of all CLOPs used in this study is their amphipathic nature, encompassing their hydrophobic and hydrophilic domains. Amphipathicity is a crucial structural property of many antiviral peptides, enabling simultaneous interactions with aqueous environments and lipid-rich viral envelopes, thus facilitating binding to viral membranes, membrane-associated glycoproteins, and protein-binding pockets [30,31,32]. Such properties have been shown to enhance peptide-mediated disruption of viral attachment, membrane fusion, and entry processes [30].

### 3.2. Molecular Properties Determining Pharmacokinetics and Drug-likeness

The physicochemical properties and drug-likeness of CLOPs were assessed utilizing SwissADME, AMDETlab 3.0, and ADMET Predictor (Table 2). The Lipinski and Pfizer rules were used to determine the drug-likeness with respect to size, solubility, and permeability. The Lipinski Rule predicts bioavailability if a compound’s MW is ≤500, nHBA ≤ 10, nHBD ≤ 5, logP (logarithm of the partition coefficient), and TPSA (topological polar surface area) are ≤5 and ≤140 Å^2^, respectively [31]. Poor bioavailability is predicted if a compound does not adhere to at least four of these rules. All CLOPs’ MWs exceeded the Lipinski rule, with the lowest predicted MW being 781.15 Daltons (Da) and nHBA above acceptable limits. P226 and P359 numbers of hydrogen bond acceptors are within the permissible standard limits, indicating good probability for increased solubility. Additionally, the TPSA ranged from 235.59 to 441.44, suggesting that these peptides will be absorbed at low concentrations in the systemic circulation. Collectively, these findings suggest that all CLOPs, rather than being administered orally, may require other routes to fully realize their therapeutic potential.

On the contrary, all CLOPs were in conformity with the Pfizer 3/75 rule, which associates toxicity to compounds with logP > 3 and TPSA < 75 Å^2^ [31]. Regarding absorption, the predictions for both parameters favor poor solubility and absorption. This, in addition to the MW, accounts for poor predictions of BBB (blood–brain barrier) penetration. The VD (volume of distribution) analysis in ADMETlab 3.0 indicates that all CLOP values are within the optimal range (0.04–20 L/kg), which is important and directly impacts dosing, safety, and efficacy. Notably, CLOPs were predicted as non-inhibitors of the major liver enzymes CYP1A2 and CYP3A4, which are responsible for metabolizing many drugs [32], predicting minimal potential of drug–drug interactions or co-administration issues with other drugs. The predicted excretion report also revealed that CLOPs had low plasma clearance rates and short half-lives, similar to what has been reported for ACV [33,34]. The toxicity assessment predicted that CLOPs are non-carcinogenic and unlikely to cause kidney damage, thus widening their therapeutic potential.

### 3.3. Machine-Learning Prioritization of CLOPs for Anti-HSV-1 Activity

**Figure 3:**
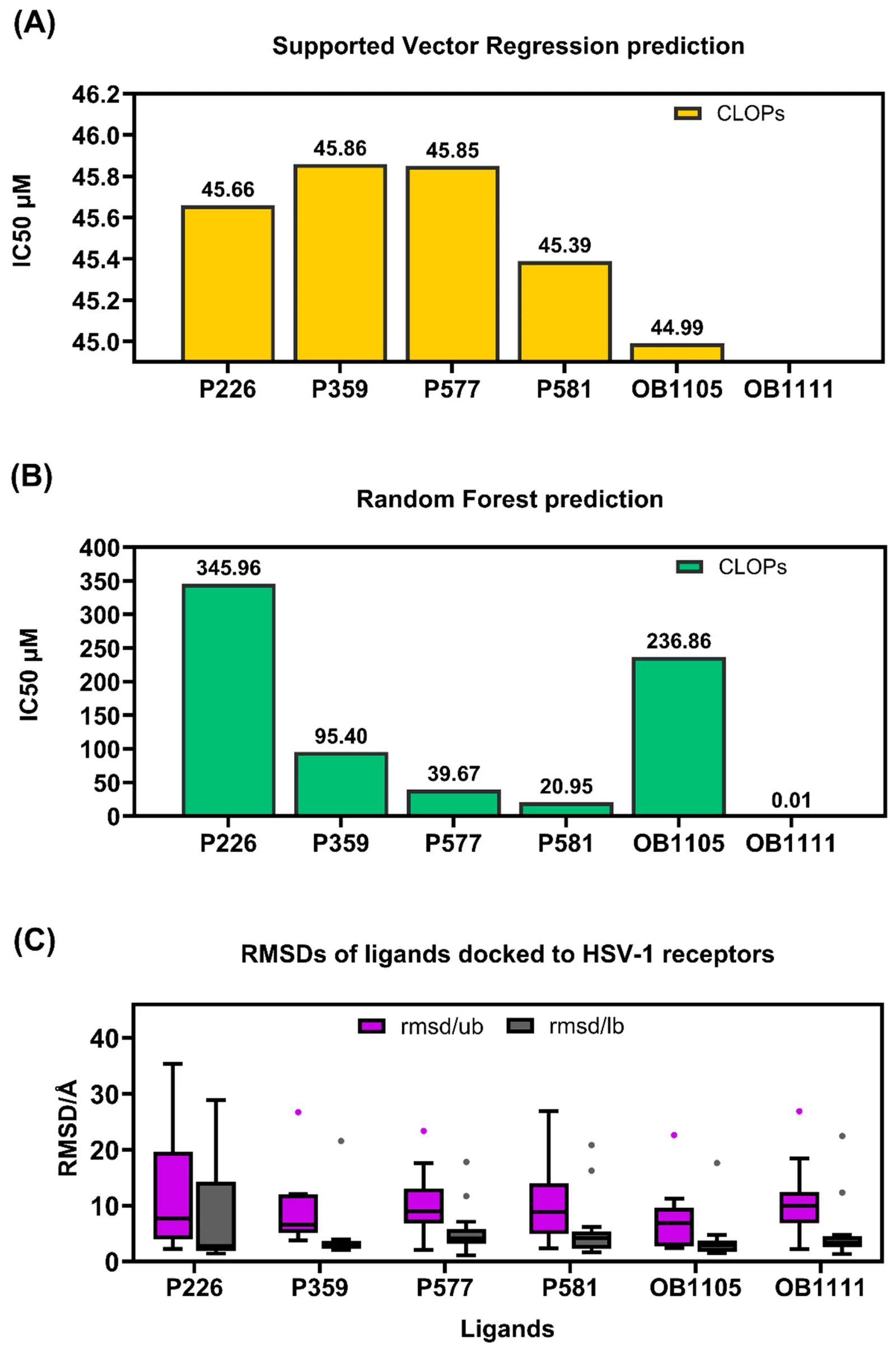
Predicted anti-HSV-1 potency of CLOPs using Support Vector Regression (**A**) and Random Forest regression (**B**). Panel (**C**) shows RMSD distributions across docking poses generated for CLOP-HSV-1 receptor pairs; these values represent docking-pose dispersion and were not obtained from molecular-dynamics simulations.

Using an SVR learning algorithm, we obtained machine-learning-predicted antiviral IC_50_ values for the CLOPs (Figure 3A). These values are computational estimates rather than experimentally observed IC_50_ measurements. The SVR model prioritized OB1111 as having the lowest predicted IC_50_. However, because the CLOPs are shorter than the peptides in the model-development dataset and the sequence descriptors do not explicitly encode lipidation or C-terminal amidation, these predictions are considered extrapolative ranking estimates. Random Forest predictions (Figure 3B) differed for several CLOPs, further emphasizing model uncertainty. OB1111 was next prioritized by both models for subsequent experimental screening. Direct quantitative validation of the predicted IC_50_ values will require multi-point concentration–response experiments to estimate empirical IC_50_ values and Hill slopes.

To further validate the reliability of the antiviral prediction, the RF algorithm was employed to predict the antiviral activities of CLOPs. This model predicted higher IC_50_ values for P226, P359, and OB1105 (Figure 3B) than the SVR, implying that CLOPs will exhibit weak antiviral activity. On the contrary, the potencies of P577 and P581 were predicted to be relatively higher, as indicated by their lower IC_50_ values of 39.67 and 20.95 µM, respectively, suggesting that lower concentrations are required to achieve the same antiviral effect, as predicted by the SVR. Interestingly, the OB1111 IC_50_ predictions from both models aligned, providing unified support for its potential as an effective anti-HSV-1 agent. The discrepancy between the two machine-learning algorithms suggests that one model may consider certain structural or physicochemical features of CLOPs that the other does not, resulting in differences in predicted IC_50_ values.

Next, we evaluated docking-pose consistency using RMSD values calculated across the nine poses generated for each CLOP-receptor docking run. As shown in Figure 3C, these RMSD distributions reflect the degree of variation among predicted docking poses and do not represent molecular dynamics trajectories. OB1105 exhibited the lowest RMSD variability, indicating a comparatively consistent predicted binding orientation, whereas P226 showed the greatest pose dispersion. OB1111 showed lower docking-pose variability than P581, P577, and P226. Accordingly, these data are interpreted as differences in docking-pose consistency rather than as direct evidence of dynamic or time-resolved complex stability.

### 3.4. Dataset Quality Assessment

The predictive performance of the machine-learning algorithms was assessed using the 683-peptide model-development dataset and the independent 76-peptide external-validation dataset. The model-development peptides ranged from 8 to 38 amino acids in length, with an average length of 22.07 amino acids and an average pIC_50_ of 5.471 (Table 3). Sequence characterization generated 578 numerical descriptors, including amino acid composition, dipeptide composition, physicochemical properties, and CTD descriptors. No peptide-sequence overlap was detected between the development and independent validation datasets. Because the CLOPs are 5–6 residues long, their predicted IC_50_ values represent extrapolation beyond the peptide-length range used for model development and are considered candidate-prioritization outputs.

**Table 3:** Summary statistics of the (**A**) antiviral peptide dataset and (**B**) data quality assessment following preprocessing.

(**A**)	
Metric	Result
Model-development peptides	683
Independent external-validation peptides	76
Total source records before within-set duplicate handling	759
Shared sequences between development and validation sets	0
Mean peptide length (model-development set)	22.07 aa
Median peptide length (model-development set)	20 aa
Peptide-length range (model-development set)	8–38 aa
Mean IC_50_ (model-development set)	37.334 µM
Mean pIC_50_ (model-development set)	5.471
(B)	
Check	Result
Training peptides	683
Independent validation peptides	76
Shared sequences	0
Conflicting duplicates	0
**Total descriptors**	578

Abbreviations: aa: amino acid residues.

### 3.5. Ten-Fold Cross-Validation Performance

The predictive performance of four machine-learning algorithms was first evaluated using ten-fold cross-validation (Table 4). Among the evaluated models, Random Forest exhibited the strongest internal predictive performance, achieving a PCC of 0.751, a Spearman correlation of 0.690, a reported squared predictive statistic of 0.561, RMSE of 0.883, and MAE of 0.672. SVR demonstrated comparable performance (PCC = 0.743), whereas Gradient Boosting showed the lowest predictive accuracy.

### 3.6. Independent External Validation

Model generalizability was subsequently assessed using an independent dataset of 76 antiviral peptides. In contrast to the internal validation results, SVR demonstrated the highest predictive performance, achieving a PCC of 0.692, a Spearman correlation of 0.572, RMSE of 0.839, and MAE of 0.651 (Table 5). Although Random Forest produced the highest cross-validation performance, its external PCC decreased to 0.625, suggesting a moderate degree of model overfitting. Overall, SVR exhibited the greatest robustness and generalization capability.

### 3.7. Leave-One-Out Cross-Validation

To further evaluate model robustness, LOOCV was performed using Ridge regression with the identical descriptor-processing pipeline. Ridge regression achieved a PCC of 0.671, a Spearman correlation of 0.591, a reported squared predictive statistic of 0.434, an RMSE of 1.002, and an MAE of 0.766 (Table 6). Exhaustive LOOCV was not performed for the nonlinear ensemble models because it required repeated fitting of 683 independent models, making the computation prohibitively expensive. Therefore, a shuffled ten-fold cross-validation and independent external validation were considered more appropriate for evaluating ensemble learning algorithms. Additional validation details can be found in Supplementary Figures S3–S8 and Supplementary Table S3.

### 3.8. Molecular Docking Against HSV-1 Glycoproteins

**Figure 4:**
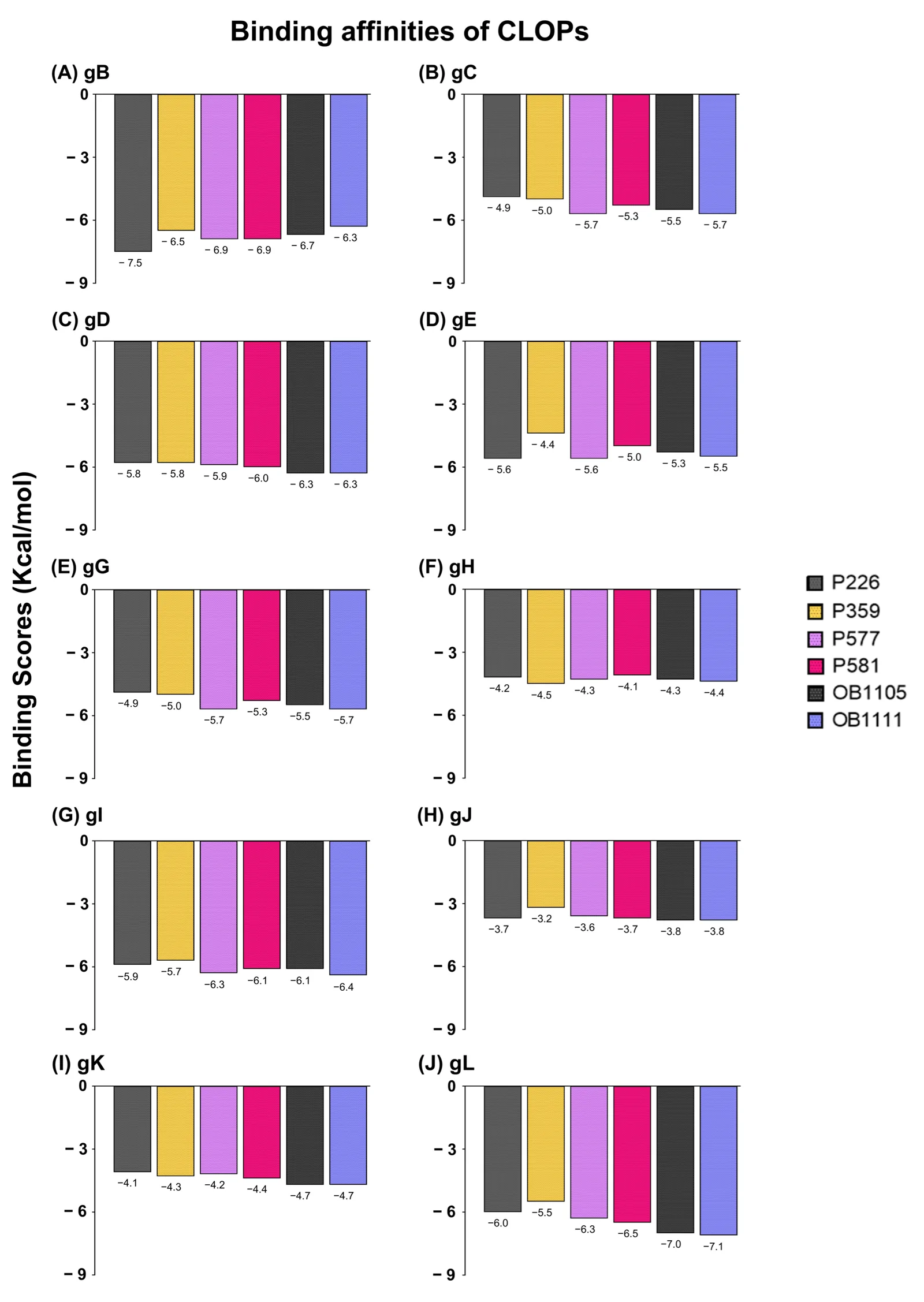
Comparative predictive binding scores for CLOPs against HSV-1 glycoproteins. The AutoDock/PyRx workflow was used to generate the docking scores, as illustrated for gB (**A**), gC (**B**), gD (**C**), gE (**D**), gG (**E**), gH (**F**), gI (**G**), gJ (**H**), gK (**I**) and gL (**J**).

Docking studies were conducted to determine the predicted binding affinities between the six CLOPs and HSV-1 glycoproteins (gB, gC, gD, gE, gG, gH, gI, gJ, gK, and gL) using Auto-Dock/PyRx-based docking [26]. Nine poses were generated for each CLOP/glycoprotein pair, and the best-scoring pose was retained for comparative analysis. Docking scores ranged from −7.5 to −3.2 kcal/mol (Figure 4). P226 showed its most favorable predicted docking scores with gB (−7.5), gL (−6.0), gI (−5.9), and gD (−5.8). P359 predictions were gB (−6.5), gD (−5.8), gI (−5.7), and gL (−5.5). P577 scores were gB (−6.9), gI (−6.3), gL (−6.3), and gD (−5.9). P581 docked with gB (−6.9), gL (−6.5), gI (−6.1), and gD (−6.0). For OB1105, scores ranged from gL (−7.0), gB (−6.7), gD (−6.3), and gI (−6.1); and for OB1111, with gL (−7.1), gI (−6.4), gB (−6.3), and gD (−6.3). Scores around −3 to −4 kcal/mol were interpreted as weak-to-modest predicted interactions, and docking scores were used for relative comparison within this computational screen.

CLOPs were subjected to further docking analysis to assess the binding orientation and residue interactions at the docking complex, as presented in the 2D and 3D structural models. HSV-1 gB and gD, both of which are important drivers of HSV-1 attachment and entry, were the preferred selected receptors. We focused our analysis on CLOPs that were predicted to bind most strongly to gB (P226 and P577) and gD (OB1105 and OB1111). Our results show the involvement of key interactions, such as alkyl interactions and individually weak interactions, that exert a substantial effect when acting collectively, typically stabilizing molecular structures [35]. Hydrogen bonds, which contribute to the overall stability of the docked complex [36], and electrostatic interactions, the type of complex interaction exerted by charged ligands that aids with repulsion and attraction [37] were also observed at the docking complex.

**Figure 5:**
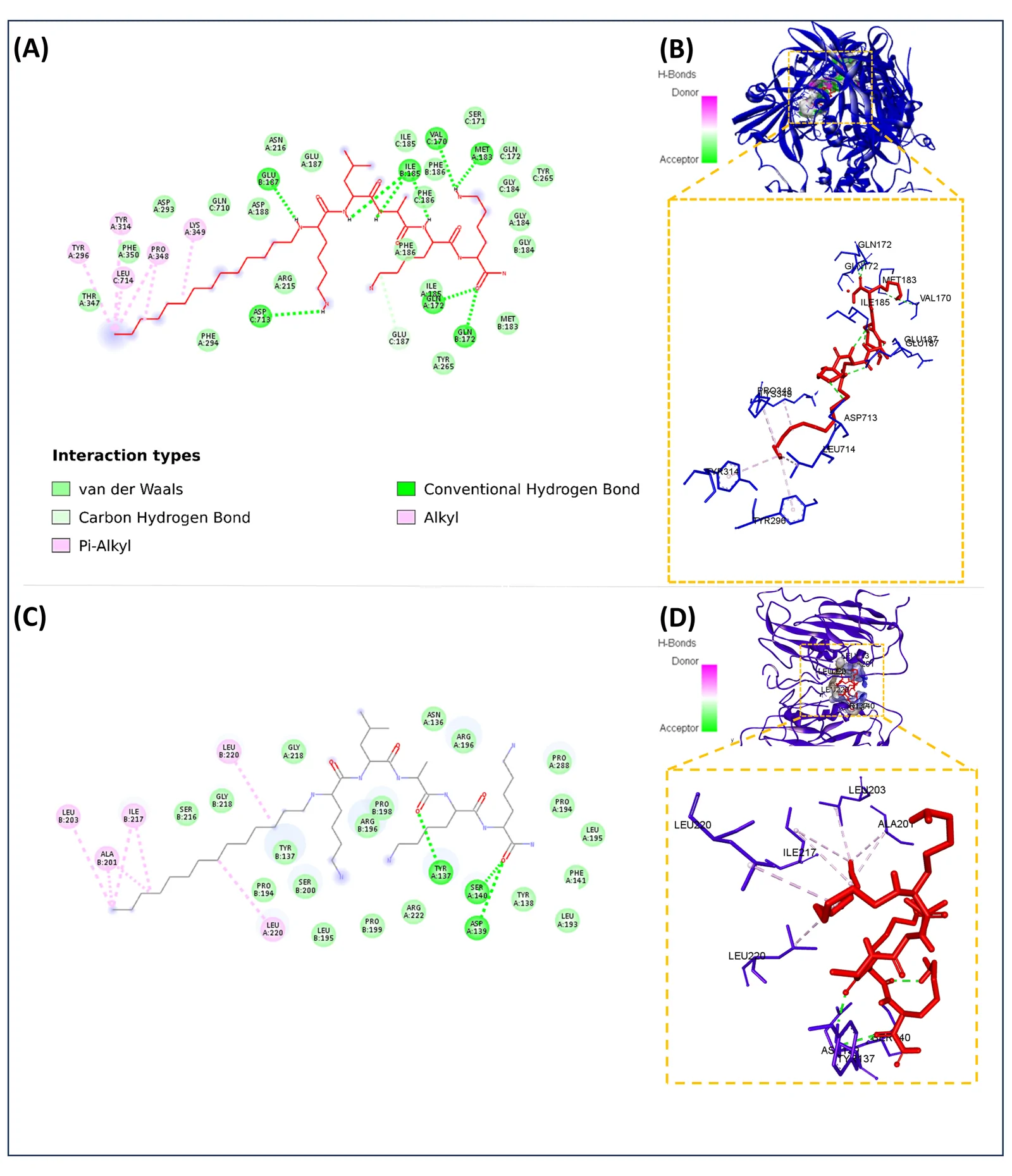
Diagrams of the 2D and 3D structures of CLOP P226, along with its interactions with HSV-1 glycoproteins B (**A**,**B**) and D (**C**,**D**). Diagrams are uniformly colored and display the interactions, amino acid residues, and CLOP/glycoprotein complex. The red ligand represents P226, the blue represents the glycoprotein, and the yellow rectangle frames the docking complex.

Specifically, we show in Figure 5 that P226 interacts with gB via alkyl interactions with Tyr296, Tyr314, Leu714, Pro348, Lys349, a carbon-hydrogen bond with Glu187, and conventional hydrogen bonds with Asp713, Glu187, Ile185, Val170, Met183, Gln172. When P226 was docked to gD, we observed alkyl interactions with Leu203, Leu220, Ala201, and Ile217, as well as conventional hydrogen bonds with Tyr137, Ser140, and Asp139.

**Figure 6:**
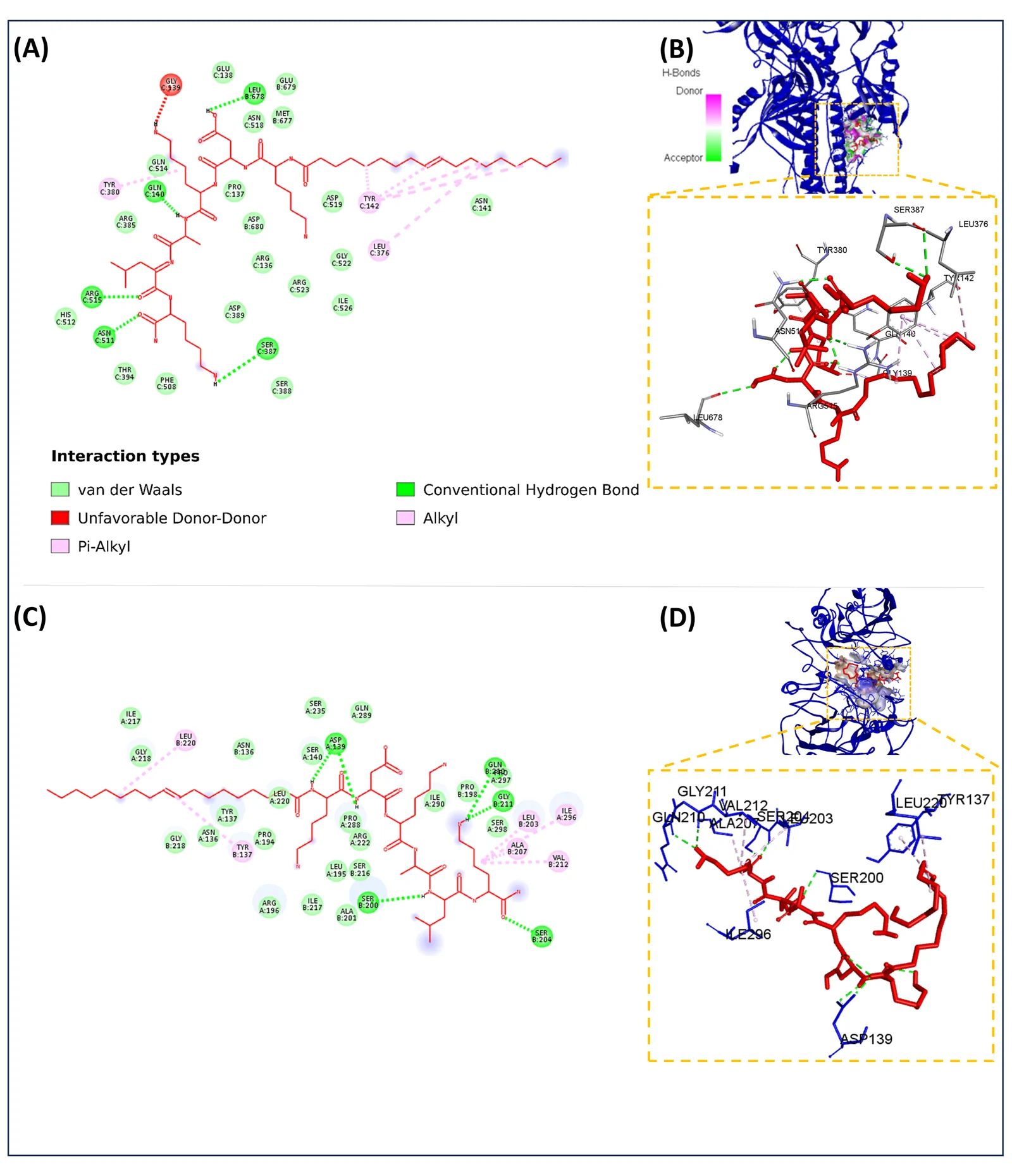
Diagrams of 2D and 3D structures of P577 interactions with HSV-1 glycoproteins B (**A**,**B**) and D (**C**,**D**). The uniform color scheme displays the interactions, amino acid residues, and CLOP/glycoprotein complex. The red ligand represents P577, the blue represents the glycoprotein, and the yellow rectangle frames the docking complex.

P577/gB interaction involves alkyl interactions with Tyr380, Tyr142, Leu376, conventional hydrogen bonds with Leu678, Gln140, Arg515, Asn511, and Ser387, and unfavorable donor–donor bonds with Gly139. When docked to gD, we observed alkyl interactions with Leu220, Leu203, Tyr137, Ala207, Val212, and Ile296, and conventional hydrogen bonds with Asp139, Ser200, Ser204, Gln210, and Gly211 (Figure 6).

**Figure 7:**
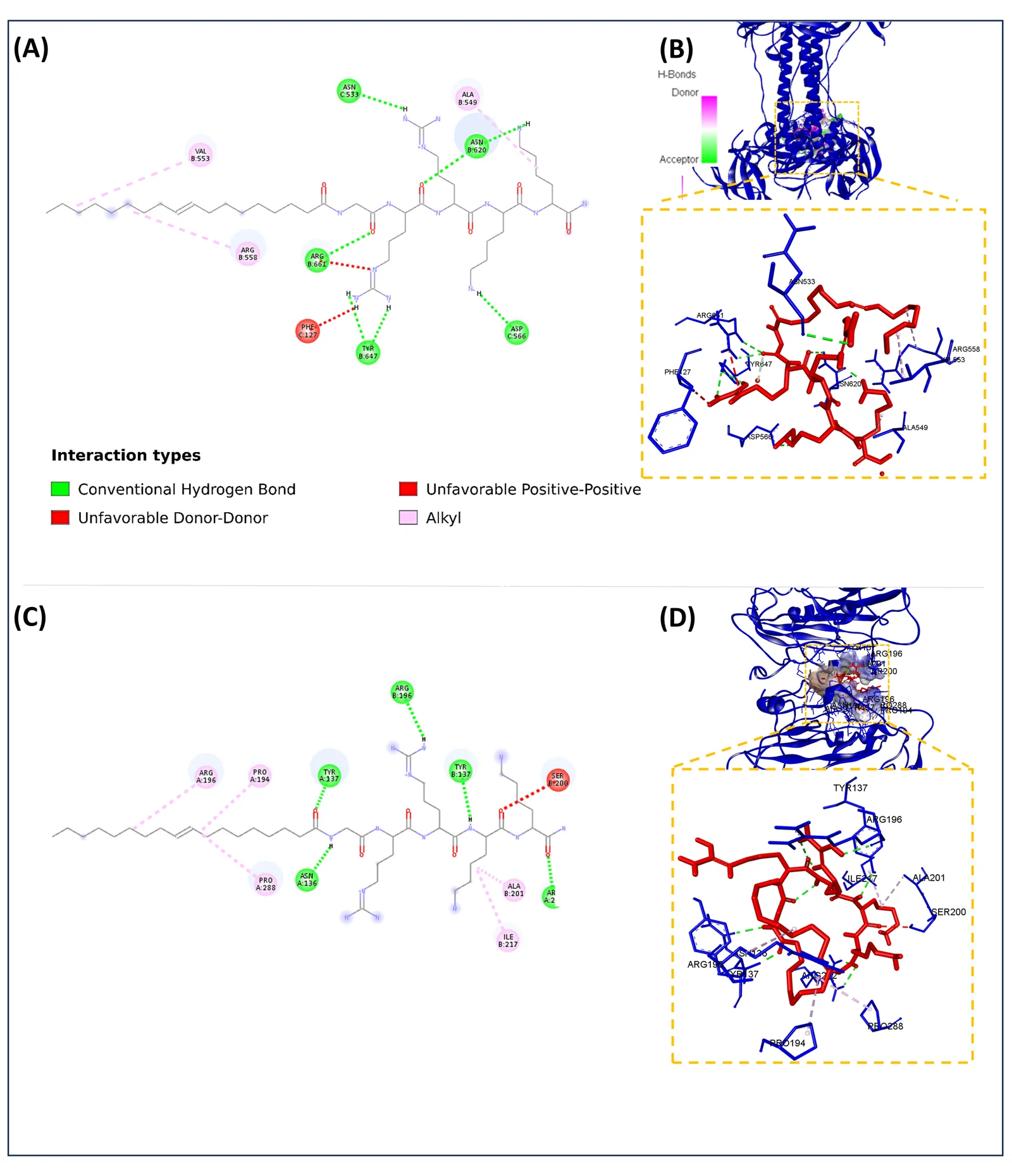
Diagrams of 2D and 3D structures of OB1105 interactions with HSV-1 glycoproteins B (**A**,**B**) and D (**C**,**D**). Diagrams are uniformly colored and display the interactions, amino acid residues, and CLOP/glycoprotein complex. The yellow rectangle frames the docking complex, which consists of OB1105 (red) and the glycoprotein (blue).

The 2D and 3D pose of the OB1105 interaction with gB shows alkyl interactions with key amino acid residues, including Val553, Arg558, Ala549, and conventional hydrogen bonds with Asn533, Asn620, Arg661, Tyr647, and Asp566, as well as an unfavorable donor–donor bond with Phe127. Alkyl interactions with Arg196, Pro194, Pro288, Ala201, and Ile217, conventional hydrogen bonds with Arg196, Arg222, Tyr137, and Asn136, as well as an unfavorable donor–donor bond with Ser200, were observed in OB1105 interaction with gD (Figure 7).

**Figure 8:**
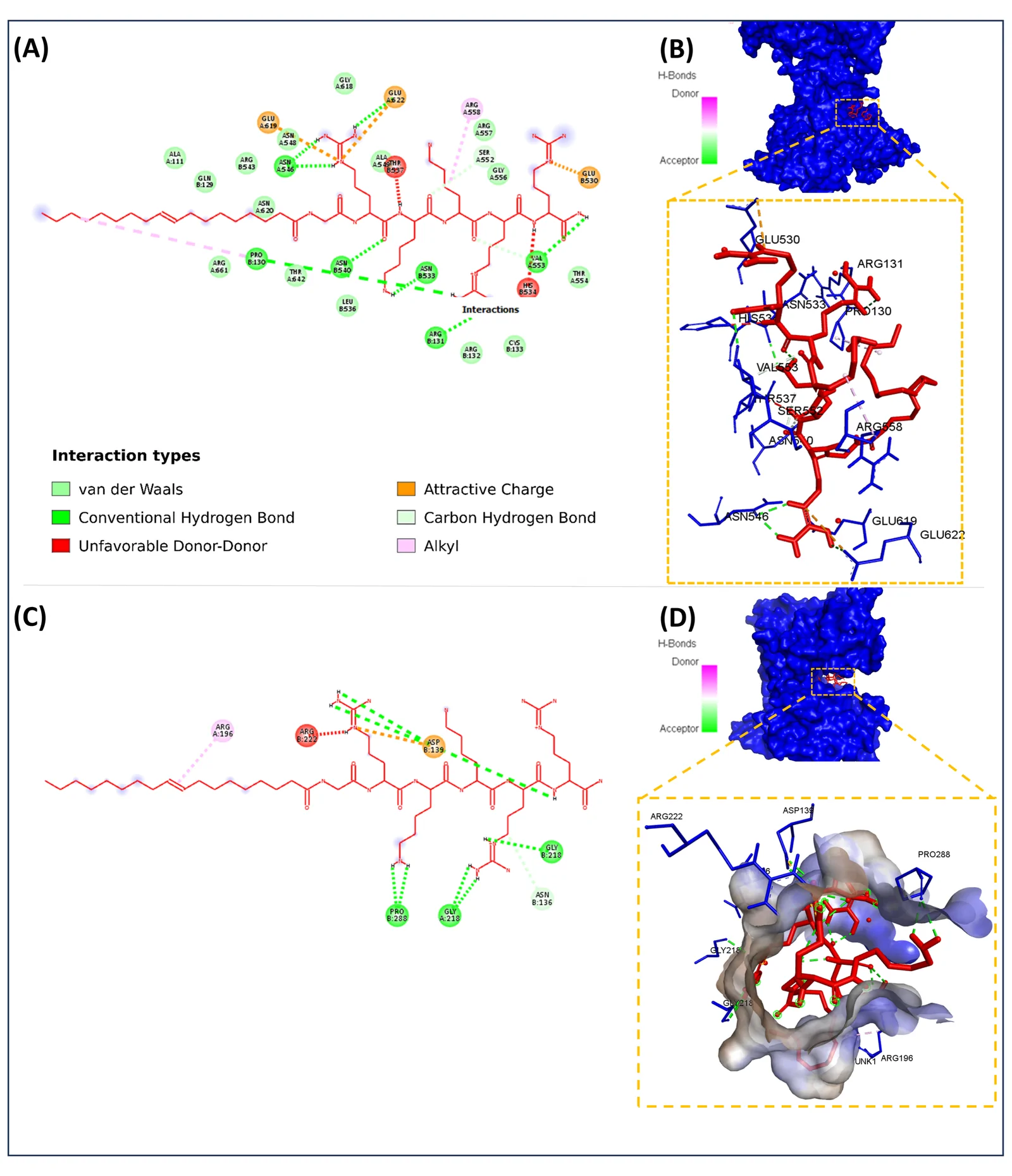
Diagrams of 2D and 3D structures of OB1111 interactions with HSV-1 glycoproteins B (**A**,**B**) and D (**C**,**D**). Diagrams are uniformly colored and display the interactions, amino acid residues, and CLOP/glycoprotein complex. The red ligand represents OB1111, the blue represents the glycoprotein, and the yellow rectangle frames the docking complex.

As depicted in Figure 8, OB1111 interacts with gB via multiple essential amino acid residues. Key amongst them includes an attractive charge with Glu619, Glu622, and Glu530, an alkyl interaction with Arg558, a carbon-hydrogen bond with Ser552, and conventional hydrogen bonds with Asn546, Val553, Pro130, Asn540, Asn533, and Arg131. On the contrary, the unfavorable donor–donor bonds observed with Thr537 and His534 residues indicate possible electrostatic repulsion and a reduction in stability of the residues. We expanded our evaluation to include OB1111 interaction with gD and found an attractive charge with Asp139, an alkyl interaction with Arg196, a carbon-hydrogen bond with Asn136, and conventional hydrogen bonds with Pro288 and Gly218. Notably, an unfavorable donor–donor bond was observed with Arg222.

To assess whether the strength of the binding affinity reflects the number of favorable interactions with amino acid residues, we evaluated the remaining CLOPs, P359 and P581. As observed in Supplementary Figure S1, P359/gB interaction involves alkyl interactions with ARG691, His692, Lys695, Ile694, conventional hydrogen bonds with Asp701, Thr703, Leu700, Glu704, and unfavorable donor–donor bonds with Tyr702. Contrastingly, when P359 interacted with gD, there were alkyl interactions with Pro198, Tyr137, Arg196, Ala201, Ile217, and Ile290, conventional hydrogen bonds with Asp139, Ser200, Ser140, and Arg222, and a carbon-hydrogen bond with Gly218. We observed alkyl interactions with Leu714, Ile719, Pro348, Lys349, a carbon-hydrogen bond with Asp713, and conventional hydrogen bonds with Glu187, Phe186, Met183 in P581/gB interaction. We also observed alkyl interactions with Pro288 and Pro198, along with the conventional hydrogen bonds with Asp139, Tyr137, Pro194, Arg196, Gly218, and Ser140 when P581 interacts with gD (Supplementary Figure S2).

We next correlated binding scores (Figure 4) with the number of interactions and interacting amino acid residues at the CLOP/glycoprotein docking complexes (Figure 4, Figure 5, Figure 6 and Figure 7 and Supplementary Figures S1 and S2) and found that CLOPs with fewer interactions and interacting amino acid residues did not necessarily have the lowest binding affinities and vice versa. Collectively, the findings from the docking score suggest that each CLOP docked to each glycoprotein is dependent on its amino acid sequence, as well as the quality, position, and strength of the interactions.

### 3.9. In Vitro Assessment of CLOPs Cytotoxicity

**Figure 9:**
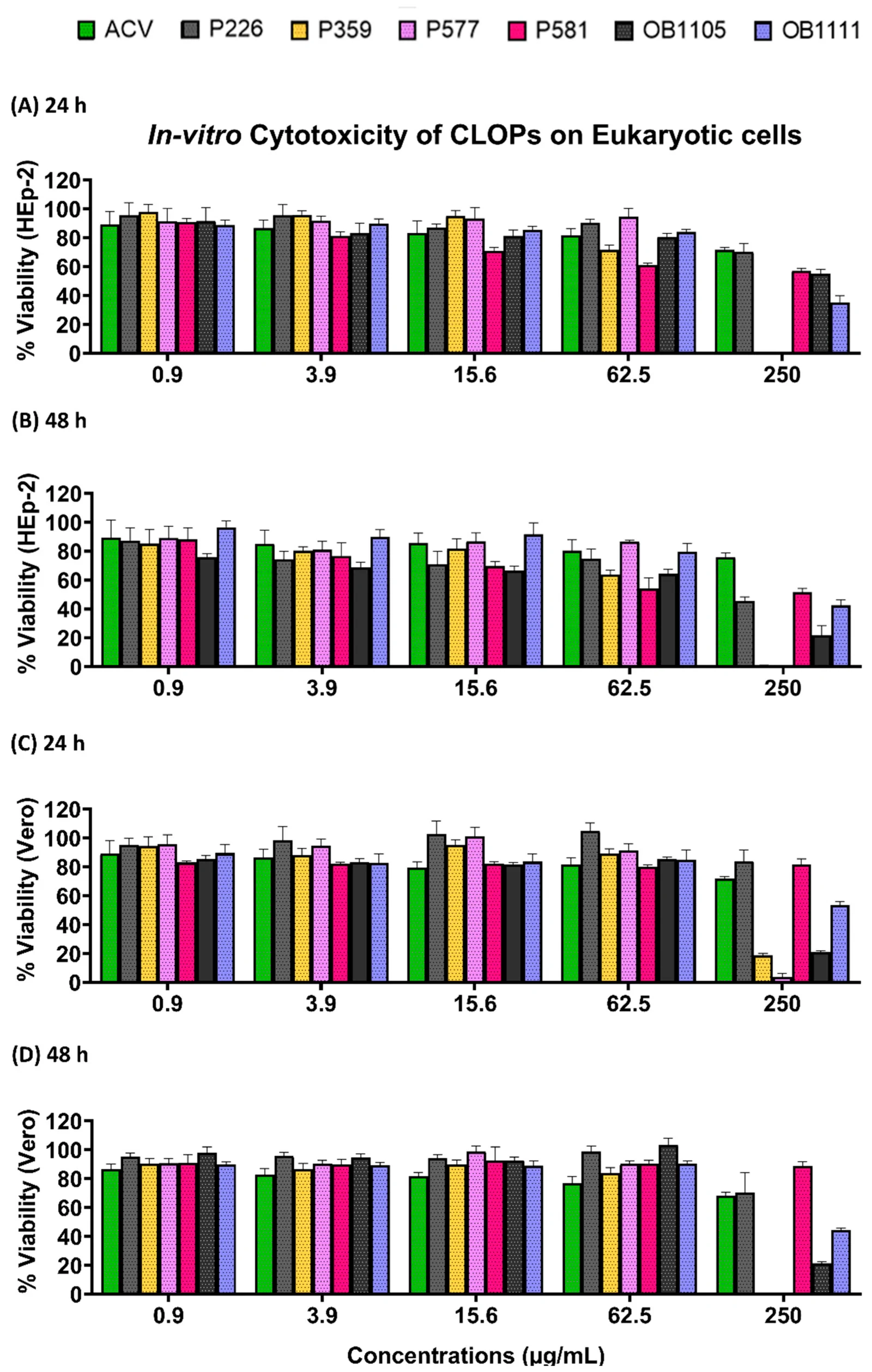
Cytotoxic effects of six CLOPs on eukaryotic cells. HEp-2 cells (5 × 10^4^/well) were treated with increasing concentrations of each CLOP (0.9 to 250 µg/mL), and cell viability was measured at 24 h (**A**) and 48 h (**B**) using the MTT assay (see Section 2). The effects of various concentrations of CLOPs (0.9 to 250 µg/mL) on the viability of Vero cells (5 × 10^4^/well) were also measured at 24 h (**C**) and 48 h (**D**) using the MTT assay. Acyclovir (ACV) served as the positive control. Data represent the mean ± SD from one of three separate experiments, each run in quadruplicate.

Assessing cytotoxicity is an important initial step in evaluating cellular tolerability. Accordingly, the cytotoxicity of CLOPs was evaluated using HEp-2 and Vero cells (Figure 9A–D) to assess their effects on the viability of distinct epithelial cell lines. Cells were incubated with increasing concentrations of CLOPs or ACV (0.9–250 µg/mL) and subjected to the MTT assay for 24 and 48 h. As shown in Figure 9A, HEp-2 cells retained ≥90% viability after 24 h of treatment with ACV, P226, P359, P577, OB1105, and OB1111 at concentrations below 250 µg/mL. P581 exerted a concentration-dependent effect on the cells’ viability. Cell viability, which was ~90% at the lowest concentration (0.9 µg/mL), progressively declined to ~60% at the highest concentrations (62.5–250 µg/mL). At the 48 h time-point, slight decreases in viability were observed with P226, P359, and OB1105 treatments, whereas cell viability remained consistent after ACV, P577, and P581 treatments. Notably, the viability of HEp-2 cells was enhanced after 48 h of exposure to OB1111 (Figure 9B). Vero cells retained ≥90% viability after 24 h of treatment with P226, P359, P577, and OB1111 at concentrations below 250 µg/mL. Cells maintained a consistent viability of ≥80% at all tested concentrations after 24 h of exposure to ACV and P581. Similar cell viability was observed at concentrations (0.9–62.5 µg/mL) after OB1105 treatment, but progressively declined to ~20% at 250 µg/mL (Figure 9C). Notably, at concentrations below 250 µg/mL, cell viability was slightly decreased after 48 h of exposure to ACV but increased after 48 h of exposure to CLOPs (Figure 9D).

### 3.10. CLOPs Displayed Anti-HSV-1 Activity

**Figure 10:**
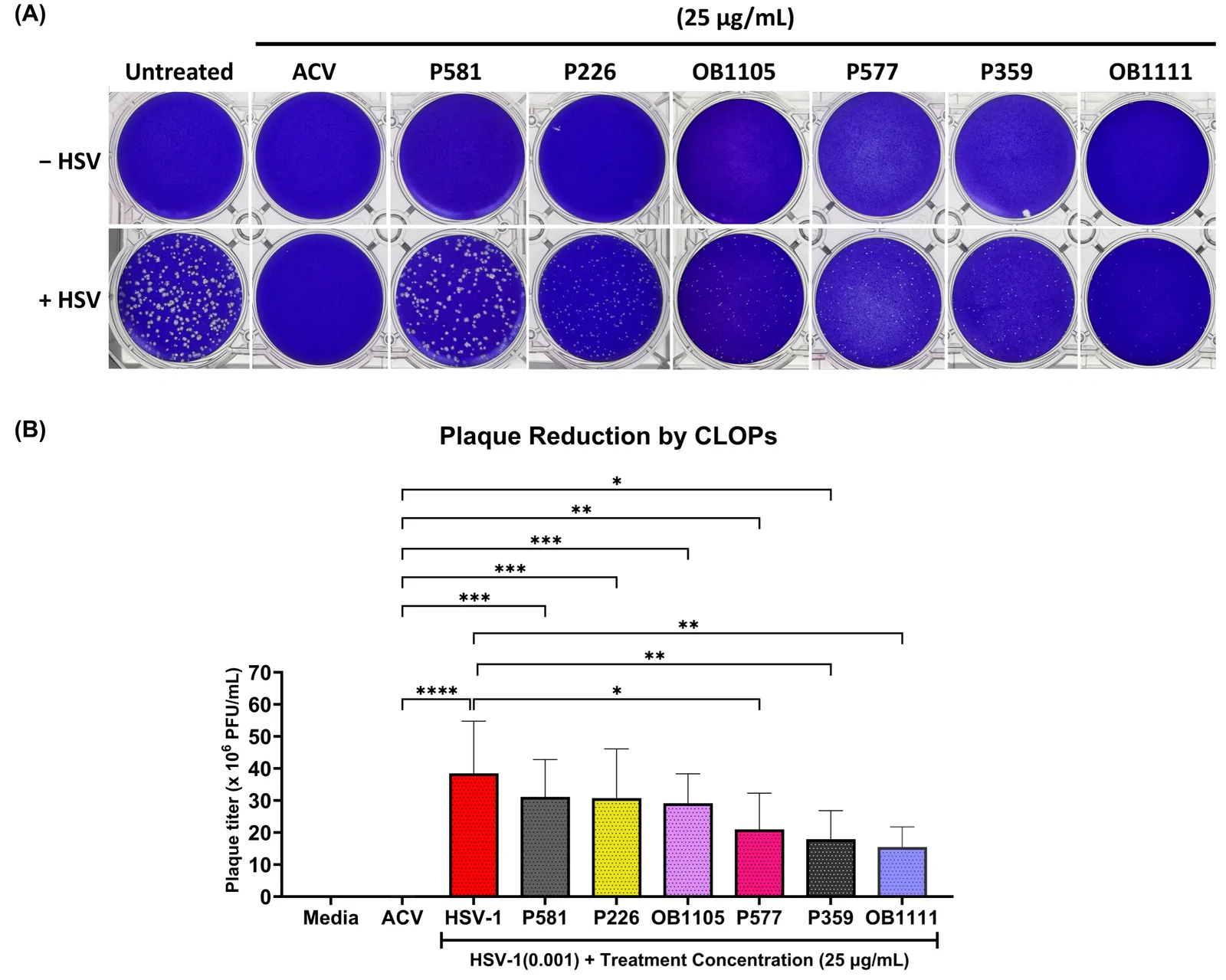
CLOPs inhibited HSV-1 plaque formation and reduced plaque sizes. Vero cells (1 × 10^6^/mL) were infected with HSV-1 (MOI of 0.001), followed by treatment with CLOPs (25 µg/mL), and subjected to the plaque reduction assay. Plaque visualization and enumeration were conducted at 48 h. Representative images of Vero cells exposed to HSV-1 and treated with CLOPs (**A**). Acyclovir (ACV) served as the positive control. The mean HSV-1 virus titers from three separate experiments were calculated and graphed (**B**). Data are presented as mean ± SD (*n* = 3 independent experiments). Statistical significance was performed using one-way ANOVA, followed by Šidák multiple comparison test at **** *p* < 0.0001, *** *p* < 0.001, ** *p* < 0.01, * *p* < 0.05.

The in silico modeling predictions of CLOPs possessing antiviral activity warranted testing their anti-HSV-1 effects using the plaque reduction assay. The primary objective of this study was to screen CLOPs at a single concentration to identify the most promising candidate for subsequent studies. Optimization studies were first conducted to select the optimal MOI, CLOPs concentration, and time-point for plaque formation. Vero cells were infected with HSV-1 (MOI of 0.001) and treated with 25 μg/mL of each CLOP. Plaque formation was visualized and enumerated at 48 h post-infection. As expected, the untreated HSV-1 control had the highest number of plaques (Figure 10A). The positive antiviral control, ACV, produced near-complete plaque suppression. When infected cells were exposed to P581, plaque size remained unaltered and was similar to that of the untreated HSV-1 control; however, plaque counts were decreased. CLOPs P226, OB1105, P577, P359, and OB1111 inhibited plaque formation, and plaque sizes were visually smaller than those formed in the untreated HSV-1 control. Ranked in ascending order, CLOP P581, P226, OB1105, P577, and P359 reduced HSV-1 plaque counts, resulting in approximately 19%, 20%, 24%, 46%, and 54% reductions in viral titer, respectively, compared to the untreated HSV-1 control (Figure 10B). OB1111 showed the greatest plaque inhibition rate, resulting in ~60% reduction in viral titer compared with the untreated HSV-1 control. The representative plaque images also suggest treatment-associated differences in plaque morphology and apparent plaque size; however, plaque area was not quantitatively measured in the present study.

Because this experiment was designed as a single-concentration screening assay, it was not used to experimentally determine CLOP IC_50_ values or to validate the absolute IC_50_ rankings predicted computationally in Figure 3A,B. Future studies will incorporate image-based plaque-area measurements, together with full multi-point concentration–response analysis, to determine empirical IC_50_ values.

### 3.11. OB1111 Suppresses HSV-1 Attachment

**Figure 11:**
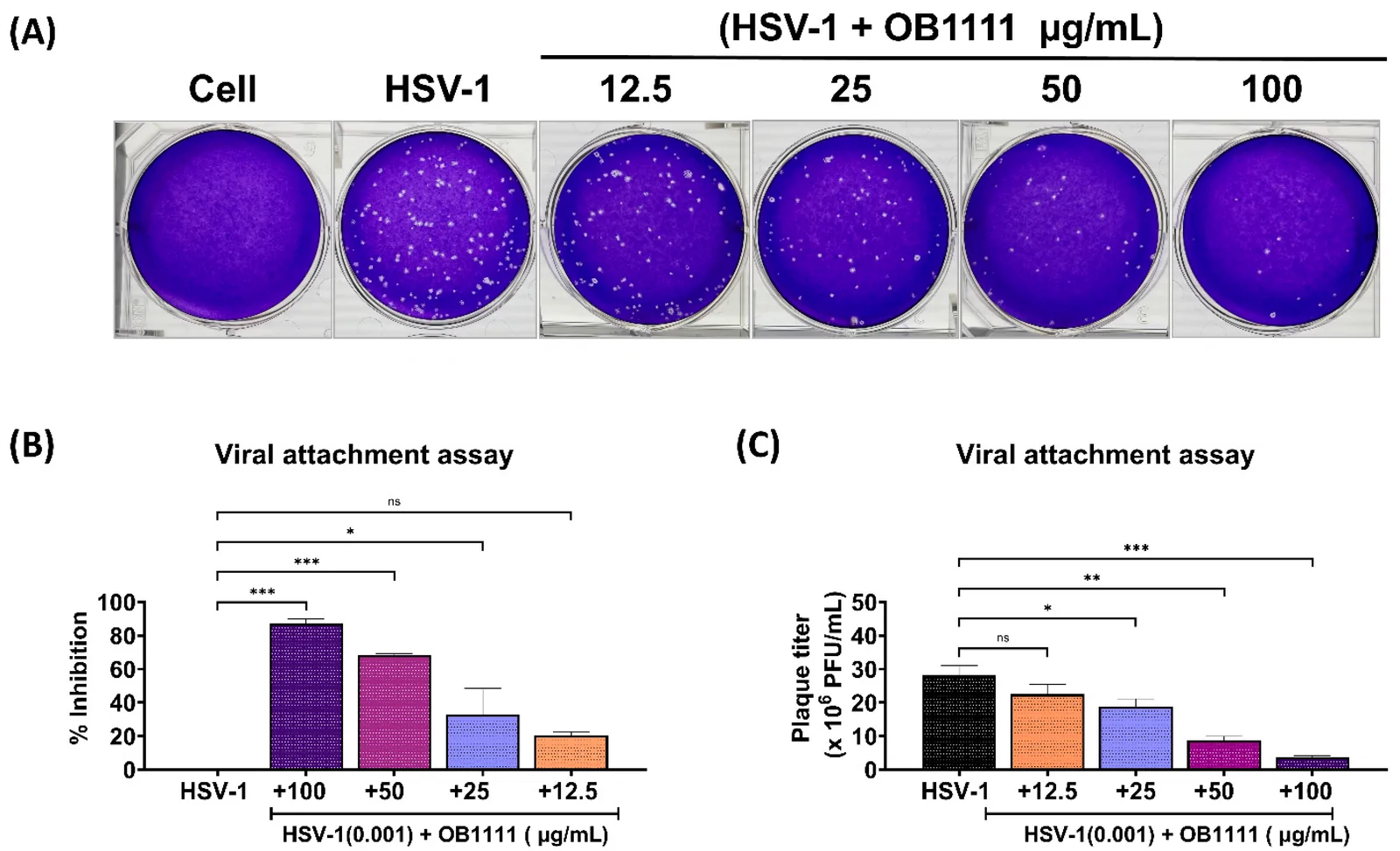
Concentration-dependent effect of OB1111 on HSV-1 plaque-forming infectivity during an early-stage attachment assay. Vero cells (1 × 10^6^/mL) were subjected to simultaneous OB1111 (12.5–100 µg/mL)/HSV-1(MOI of 0.001) administration at 4 °C, followed by a temperature shift to 37 °C to permit entry (**A**). Plaques were visualized and enumerated after 48 h, and the percent inhibition (**B**) and titers (**C**) were determined. Data are presented as the mean ± SD (*n* = 2 independent experiments). One-way ANOVA was used to evaluate treatment effects; Bartlett’s test was used to assess homogeneity of variances at *** *p* < 0.001, ** *p* < 0.01, * *p* < 0.05.

OB1111 was selected as the lead candidate because it showed comparatively favorable predicted docking interactions with several HSV-1 glycoproteins and was the top-ranked antiviral CLOP by both machine-learning models. OB1111 also exhibited the strongest antiviral activity among the six CLOPs evaluated (Figure 3, Figure 4 and Figure 10). These convergent observations, particularly its predicted ability to bind to HSV-1 entry glycoproteins, prompted evaluating OB1111’s effect on the early stage of viral attachment to cells. As such, experiments were conducted under specific screening conditions. Cells were exposed to HSV-1 in the presence of OB1111 at 4 °C, a condition that favors attachment but not cell entry. Cells were washed to remove unbound viruses, kept at 37 °C to permit entry, incubated at 37 °C for 48 h, and the resulting plaques were enumerated. We observed that OB1111 exhibited concentration-dependent antiviral activity under attachment-associated conditions as compared with the HSV-1 control (Figure 11A). HSV-1 infectivity was reduced, as evidenced by an increase in percent inhibition (Figure 11 B) and a decrease in virus titer (Figure 11 C) with increasing peptide concentrations. Overall, the data suggest that OB1111 may interfere with the early stages of the HSV-1 life cycle.

## 4. Discussion

HSV-1, a well-understood human pathogen, remains incurable, constantly infecting new hosts. It relies on glycoproteins for entry into host cells, and for other essential processes that facilitate its propagation and persistence in humans [9]. This makes them attractive targets for the development of novel alternative therapeutics. AMPs have emerged as promising alternative therapeutics for treating HSV-1 infections, and several have demonstrated activity against HSV-1 glycoproteins [8,17,38,39]. In this study, we employed in silico computational and in vitro experimental approaches to assess the antiviral effects of six novel CLOPs against HSV-1. All of the CLOPs are cationic and amphipathic, comprising a N-terminal lipid moiety, which is strongly hydrophobic, and a C-terminal amidation, which confers its hydrophilicity. Hydrophobic residues are integral to AMPs’ specificity and membrane insertion [40]. The hydrophilic nature of CLOPs is essential for maintaining their solubility in aqueous solution. Such features are known to facilitate electrostatic interactions with viral envelopes and glycoproteins, thereby contributing to antiviral activity [41,42,43].

In silico ADMET and drug-likeness evaluations provided an initial computational screen into the pharmacokinetic and toxicology profiles of the CLOPs. The Lipinski’s rule, a rule commonly used for solubility and bioavailability [44], was a determinant of CLOPs’ drug-likeness. The CLOPs did not conform to multiple Lipinski criteria, as is not unexpected for peptide-like molecules, which typically have MWs that exceed the membrane-permeability limits set by the Lipinski rule [45]. A notable fraction of orally administered FDA-approved small-molecule drugs violate at least one criterion of this rule [46]. Roskoski et al. (2023) successfully outlined Dabrafenib, along with 29 other FDA-approved drugs that violate Lipinski’s rule despite maintaining relevant clinical bioavailability [47]. More practically, the MW of Rybelsus^®^, an orally administered FDA-approved peptide-based drug used to treat type 2 diabetes, exceeds the Lipinski rule by ~3613.58 Da [48,49]. This means that while this rule sets the foundational framework for initial drug screening, its violation does not always indicate poor bioavailability or clinical relevance. When these rules are violated and bioavailability is compromised, alternative routes of administration could be explored for compounds in this category. Topical application is an ideal approach because it delivers the treatment directly to the site of infection, resulting in rapid therapeutic action [50]. This approach is particularly relevant for infections such as HSV-1, which causes multifocal mucosal lesions [51]. They are most effective at minimizing lesion duration and discomfort when used at the onset of infection [50] and are typically formulated as topical applications [52], providing individuals with the flexibility to select a formulation most suitable for their skin type.

The Pfizer 3/75 rule was the second key determinant used for drug-likeness rating. All CLOPs complied with this rule, indicating favorable toxicity predictions, as validated by the MTT cytotoxicity assay (Figure 9). CLOPs ADMET profiling displays favorable predictions for parameters such as volume of distribution, metabolism, and toxicity. Absorption, BBB permeability, and excretion predictions were poor (Table 2B). However, counteractive techniques such as transformative oral delivery, the use of absorption and permeation enhancers, exploration of alternative administration routes, and chemical modification of peptides using molecular techniques can address these limitations [53,54].

The computational docking analysis predicted potential binding of CLOPs to the HSV-1 glycoproteins evaluated (Figure 4), with the most favorable scores frequently observed for gD, gB, gL, and gI. Residue-level inspection identified predicted electrostatic, alkyl, carbon-hydrogen, and conventional hydrogen-bond contacts that may contribute to the relative docking scores. However, docking scores are model-dependent estimates of pose favorability and do not establish biochemical binding, target specificity, or inhibition. Differences in the number of predicted residue contacts did not consistently track with docking score, indicating that pose geometry and the scoring function contribute substantially to the ranking. For example, OB1111 had a lower affinity to gB than P359 (Figure 4A), despite its interaction with more amino acid residues than P359 (Figure 8A and Figure S1A). Another scenario involves the interaction of OB1105 and OB1111 with gD, in which both had the same docking scores (Figure 4C) despite fewer interactions and amino acid residues in the OB1105/gD interaction than in the OB1111/gD interaction (Figure 7C and Figure 8C). Values near –3 to –4 kcal/mol were classified as weak-to-modest predicted interactions. Further mechanistic confirmation will require direct binding and functional entry/fusion assays.

Results from the MTT experiments showed that CLOPs were not toxic to HEp-2 and Vero cells at concentrations below 250 µg/mL. Interestingly, we observed enhanced cell viability after 48 h of CLOPs treatment, demonstrating that they are well-tolerated by cells and have potential for broad application. While this is promising, broader toxicology and pharmacokinetic studies will be required before safety or clinical suitability can be assessed.

In the present study, all CLOPs were predicted to inhibit HSV-1 infection, with a range of IC_50_ values across two independent machine-learning algorithms, as shown in Figure 3A,B. Because variability was observed in the ranking of CLOPs’ antiviral efficacy between the SVR and RF machine-learning algorithms, model validation was conducted to evaluate the predictive performance and robustness of the models. Both machine-learning algorithms demonstrated a moderate-to-strong relationship between peptide sequence descriptors and antiviral activity. RF achieved the highest internal predictive performance during cross-validation, whereas SVR exhibited superior generalization on the independent dataset. The reduction in RF performance from cross-validation (PCC = 0.751) (Table 4) to external validation (PCC = 0.625) (Table 5) suggests a modest degree of overfitting, while the relatively stable performance of SVR (PCC = 0.743 vs. 0.692) indicates greater robustness for predicting antiviral peptide activity, as shown in Table 4 and Table 5. These findings demonstrate that sequence-derived descriptors provide substantial predictive information for peptide antiviral potency and support the use of SVR as the preferred model for external prediction. The RF model offers nonlinear feature interactions and a strong internal fit, whereas SVR showed better external generalization in this dataset. Nevertheless, the moderate external correlations and prediction errors indicate that these models should be viewed as prioritization tools rather than substitutes for experimentally determined dose–response IC_50_ values.

In vitro plaque reduction assays showed that CLOPs reduced HSV-1 plaque counts by ~ 19–60% and visibly reduced plaque sizes (Figure 10A,B). OB1111 demonstrated the strongest antiviral activity among the six CLOPs evaluated and was also prioritized by the computational models. The follow-up early-stage assay showed a concentration-dependent reduction in plaque-forming infectivity with OB1111, implying interference with an early phase of infection. Other studies have evaluated peptides as alternatives to current HSV-1 drugs, such as temporin B, which exhibited in vitro antiviral activity against HSV-1, possibly by impairing gB expression, which may restrict viral spreading [55]. Another study by Zaczyńska et al. (2022) assessed the antiviral properties of the 4B8M peptide in HSV-1-infected A-549 cells, which markedly reduced the HSV-1 virus titer. Similar to our results, peptide 4B8M was well-tolerated and displayed high potency against the HSV-1 [56]. Two other peptides, MEL-AM and MEL-AF, had an inhibitory effect on HSV-1 viral attachment of less than 50%. Both peptides had no effect on HSV-1 plaque size, despite suppressing cytopathic effects, as evidenced by reduced cell rounding, aggregation, and nuclear enlargement. The lack of effect on plaque size with these peptides, as observed in five of the six CLOPs tested in our study, might be due to the peptides’ effect on viral spread, and their presence or absence may indicate differences in the mechanistic action of peptides [57]. Contrary to our results, LL-37, a human cathelicidin peptide, failed to block HSV-1 activity in infected human corneal epithelial cells after post-infection administration [58]. Another peptide, Eval418, displayed weak antiviral activity against HSV-1 following pre-treatment and post-treatment applications, and strong antiviral activity when administered during the attachment phase [59]. Similarly, TL and TL1 exerted negligible inhibitory effects on HSV-1 in pre-treatment and post-treatment assays, but significant inhibitory effects when administered prior to or during infection, suggesting a tendency for prophylactic use [16]. This provides evidence for AMP’s ability to target multiple stages of the HSV-1 life cycle.

A major distinction between the CLOPs used in the present study and those referenced above is the length of their amino acid sequences. Our CLOPs are short, consisting of 5–6 amino acid residues, whereas the ones mentioned above are longer (ranging from 13 to 40 amino acid residues). This may be an added advantage, as smaller peptides can more readily cross the membrane and exhibit higher bioavailability than their larger counterparts [60]. Additionally, the antiviral activity of CLOPs could be assessed across a wider concentration range before cytotoxicity was induced, indicating greater cellular tolerance. For example, MEL-AM and MEL-AF peptides significantly reduced RAW 264.7 cell viability at 20 µg/mL after 24 h [57]. In contrast, our CLOPs significantly affected the viability of Vero and HEp-2 cells at 250 µg/mL after 24 and 48 h of exposure, indicating their non-toxicity.

The single-target nature of ACV contrasts sharply with the multimodal nature of most AMPs, potentially offering many avenues for antiviral intervention. In response to the increasing occurrence of ACV-resistant strains, AMPs’ diverse mechanisms suggest that more emphasis should be placed on investigating them as a means of addressing this challenge [61,62].

## 5. Conclusions

In this study, we employed an integrated in silico and in vitro approach to evaluate six candidate CLOPs against HSV-1. Computational predictive analyses revealed their low-risk toxicity, potential antiviral activity against HSV-1, and binding affinities for major viral glycoproteins, supported by stable interaction profiles. Experimental analyses support their non-cytotoxicity and inhibition of viral infectivity and titers, as evidenced by a reduction in plaque counts and sizes. OB1111 was the top-ranked predicted antiviral CLOP and experimentally produced the strongest antiviral activity in the plaque reduction assay, and reduced the plaque-forming efficiency of HSV-1 in the subsequent early-stage experiment in a dose-dependent manner. These findings support prioritizing OB1111 for further evaluations of its antiviral effects and mechanisms against HSV-1.

## Figures and Tables

**Table 1:** Tabulated display of the molecular features of CLOPs.

CLOP	Composition	Net Charge	Structural Features	Terminal Modifications
P226 P359 P577 P581	Pentapeptides containing three Lys, one Leu, and one Ala	+ 3 (from Lys residues)	Amphipathic, α helical	N-terminal lipidation (hydrophobic); C-terminal amide (hydrophilic)
OB1105	Pentapeptide containing two Lys, two Arg, and one Gly	+4 (from Lys and Arg residues)	Amphipathic	
OB1111	Hexapeptide containing two Lys, three Arg, and one Gly	+5 (from Lys and Arg residues)	Amphipathic	

Abbreviations: Lys: lysine; Leu: leucine; Ala: alanine; Arg: arginine; Gly: glycine.

**Table 2:** Summary of the (**A**) physiochemical and drug-likeness of CLOPs and (**B**) ADMET analysis results of CLOPs.

(**A**)												
CLOP		MW (Da)		nROT	nHBA		nHBD		LogP	TPSA	Drug-Likeness	
											Lipinski	Pfizer
P226		781.15		41	10		9		3.434	235.59	No	Yes
P359		781.15		41	10		9		3.436	235.59	No	Yes
P577		964.31		49	13		11		3.157	319.06	No	Yes
P581		896.23		46	13		11		2.801	301.99	No	Yes
OB1105		906.24		48	11		14		2.131	364.43	No	Yes
OB1111		1062.42		56	13		18		1.214	441.44	No	Yes
(**B**)												
CLOP	Absorption			Distribution		Metabolism			Excretion		Toxicity	
	Log S		Pgp Substrate	BBB -PS	VD (L/kg)	CYP1A2		CYP3A4	Half-Life	CL plasma	Carc.	Nephrotox.
P226	−3.2		Yes	No	14.117	No		No	1.0008	4.513	0.028	0.544
P359	−3.2		Yes	No	12.042	No		No	1.038	4.379	0.023	0.467
P577	−3.621		Yes	No	1.260	No		No	1.337	3.062	0.005	0.461
P581	−4.148		Yes	No	1.640	No		No	0.446	5.045	0.195	0.255
OB1105	−3.299		Yes	No	1.821	No		No	0.979	4.043	0.033	0.92
OB1111	−3.13		Yes	No	1.716	No		No	1.364	3.455	0.032	0.99

Abbreviations: CLOP: cationic lipo-oligopeptides; MW: molecular weight; nROT: number of rotatable bonds; nHBA: number of hydrogen bond acceptors; nHBD: number of hydrogen bond donors; LogP: logarithm of the partition coefficient; TPSA: topological polar surface area. Log S: logarithm of solubility; Pgp: P-glycoprotein; BBB: blood–brain barrier passage; VD: volume of distribution; CYP1A2: cytochrome P450 1A2; CYP3A4: cytochrome P450 3A4; CL plasma: plasma clearance; Carc.: Carcinogenicity; Nephrotox.: Nephrotoxicity.

**Table 4:** Ten-fold cross-validation performance of machine-learning regression models.

Model	PCC	Spearman	Squared Statistic	RMSE	MAE
Random Forest	0.751	0.690	0.561	0.883	0.672
SVR	0.743	0.683	0.545	0.898	0.649
XGBoost	0.740	0.680	0.544	0.899	0.695
Gradient Boosting	0.714	0.650	0.501	0.940	0.729

**Table 5:** Performance of machine-learning models on the independent validation dataset.

Model	PCC	Spearman	Squared Statistics	RMSE	MAE
SVR	0.692	0.572	0.432	0.839	0.651
XGBoost	0.628	0.592	0.388	0.871	0.670
Random Forest	0.625	0.539	0.374	0.881	0.658
Gradient Boosting	0.612	0.545	0.371	0.883	0.691

**Table 6:** Performance of Ridge regression during leave-one-out cross-validation.

Model	PCC	Spearman	Squared Statistic	RMSE	MAE
LOOCV	0.671	0.591	0.434	1.002	0.766

## Data Availability

The original contributions presented in this study are included in the article/supplementary material. Further inquiries can be directed to the corresponding author.

## Supplementary Materials

The following supporting information can be downloaded at https://www.mdpi.com/article/10.3390/microorganisms14102182/s1. Figure S1: Diagrams of 2D and 3D structures of CLOP P359 interactions with HSV-1 glycoproteins; Figure S2: Diagrams of 2D and 3D structures of CLOP P581 interactions with HSV-1 glycoproteins; Figure S3A,B: Distribution of peptide length and experimental pIC_50_ values after reprocessing; Figure S4: PCA of the 578 sequence-derived descriptors; Figure S5: RF: ten-fold cross-validation; Figure S6: SVR: independent external validation; Figure S7A,B: Residuals of: RF ten-fold cross-validation and SVR independent external validation; Figure S8: Top 10 RF feature-importance rankings; Table S1: List of computational tools; Table S2: Summary of the HSV-1 glycoproteins, accession numbers, and structure acquisition methods; Table S3: Statistical metrics used to evaluate the model performance; Table S4: Summary of the structural quality metrics of the predicted three-dimensional models for HSV glycoproteins generated using the SWISS-MODEL server.

## Abbreviations

The following abbreviations are used in this manuscript:

aa	Amino acid residues
AAC	Amino acid composition
ACV	Acyclovir
ADMET	Absorption, Distribution, Metabolism, Excretion, and Toxicity
Ala	Alanine
AMPs	Antimicrobial peptides
APD	Antimicrobial Peptide Database
Arg	Arginine
Asn	Asparagine
AUC	Area under the curve
AVPdb	Antiviral Peptide Database
BBB	Blood–brain barrier passage
Carc.:	Carcinogenicity
CLOPs	Cationic lipo-oligopeptides
CLplasma	Plasma clearance
CML	Chemical Markup Language
CTD	Composition-transition-distribution
CYP1A2	Cytochrome P450 1A2
CYP3A4	Cytochrome P450 3A4
Da	Dalton
DPC	Dipeptide composition
G	Glycoproteins
Gln	Glutamine
Glu	Glutamate
Gly	Glycine
GMQE	Global Model Quality Estimation
His	Histidine
HSV-1	Herpes simplex virus type 1
IC_50_	Half-maximal inhibitory concentration
Ile	Isoleucine
InChI	International Chemical Identifier
IUPAC	International Union of Pure and Applied Chemistry
Leu	Leucine
LogP	Logarithm of the partition coefficient
Log S	Logarithm of solubility
LOOCV	Leave-one-out cross-validation
Lys	Lysine
MAE	Mean absolute error
Met	Methionine
MW	Molecular weight
NCBI	National Center for Biotechnology Information
Nephrotox.	Nephrotoxicity
nHBA	Number of hydrogen bond acceptors
nHBD	Number of hydrogen bond donors
nROT	Number of rotatable bonds
PCC	Pearson correlation coefficient
PDB	Protein Data Bank
Pgp	P-glycoprotein
Phe	Phenylalanine
pI	Isoelectric point
Pro	Proline
RF	Random Forest
RMSE	Root mean square error
ROC	Receiver operating characteristic
Ser	Serine
SMILES	Simplified Molecular Input Line Entry System
SVR	Support Vector Regression
Thr	Threonine
TPSA	Topological polar surface area
Tyr	Tyrosine
Val	Valine
VD	Volume of distribution
XGBoost	Extreme Gradient Boosting
