# Supplementary material for: Proprietary Cationic Lipo-Oligopeptides Inhibit Herpes Simplex Virus-1 Infection
Source: Microorganisms. 2026 Sep 29;14(10):2182. doi: 10.3390/microorganisms14102182 (PMC13707818; doi:10.3390/microorganisms14102182)
Supplement: Supplementary file 1 [file microorganisms-14-02182-s001.zip › Supplementary Tables.pdf]

**Supplementary Table S1.**

List of computational tools

| Software | Version | Purpose | Website |
|---|---|---|---|
| **Protein Data Bank** | Current Release | Protein structure retrieval | https://www.rcsb.org |
| **PyMOL** | 2.x | Structure visualization and preparation | https://pymol.org |
| **Gene Bank** | Current Release | Reference Amino Acid Sequence retrieval | https://www.ncbi.nlm.nih.gov/gene/?term= |
| **Discovery Studio Visualizer** | 2021 | Interaction analysis | https://discover.3ds.com/discovery-studio-visualizer-download |
| **AutoDock Tools** | 1.5.7 | Protein and ligand preparation | https://autodock.scripps.edu |
| **AutoDock Vina** | 1.2.x | Molecular docking | https://vina.scripps.edu |
| **SwissADME** | Current | Drug-likeness and ADME prediction | https://www.swissadme.ch |
| **SwissModel** | Current | Homology modeling | https://swissmodel.expasy.org/ |
| **ADMETlab 3.0** | Current | ADMET prediction | https://admetmesh.scbdd.com |
| **DataWarrior** | Current | An Open-Source Program for Data Visualization and Analysis with Chemical Intelligence | https://openmolecules.org/datawarrior/ |
| **OPSIN software** | Current | Interprets systematic IUPAC nomenclature for chemistry and biochemistry and converts it into chemical structures reported as SMILES, InChI and CML | https://www.ebi.ac.uk/opsin |
| **Cellppd** | Current | Predict and design efficient cell penetrating peptides | https://bio.tools/cellppd |

**Supplementary Table S2.**

Summary of the HSV-1 glycoproteins, accession numbers, and structure acquisition methods

| Glycoprotein | Structure Source | Accession Number / PDB ID | Structure Acquisition Method |
|---|---|---|---|
| **gB** | Protein Data Bank | 3NW8 | Experimental structure downloaded from PDB |
| **gC** | GenBank | AFH78104.1 | Homology model generated using Swiss-Model |
| **gD** | Protein Data Bank | 2C36 | Experimental structure downloaded from PDB |
| **gE** | GenBank | AYG87808.1 | Homology model generated using Swiss-Model |
| **gG** | GenBank | AYG87875.1 | Homology model generated using Swiss-Model |
| **gH** | Protein Data Bank | 2LQY | Experimental structure downloaded from PDB |
| **gI** | GenBank | AAW49017.1 | Homology model generated using Swiss-Model |
| **gJ** | GenBank | AOY34336.1 | Homology model generated using Swiss-Model |
| **gK** | GenBank | AAB60558.1 | Homology model generated using Swiss-Model |
| **gL** | GenBank | AFH78105.1 | Homology model generated using Swiss-Model |
| **gM** | GenBank | AOY34334.1 | Homology model generated using Swiss-Model |
| **gN** | GenBank | AOY34292.1 | Homology model generated using Swiss-Model |

**Supplementary Table S3.**

Formulas and statistical metrics used to evaluate the model performance

Pearson correlation coefficient was calculated as:

$$PCC = \frac{\sum (y_i - \bar{y})(\hat{y}_i - \bar{\hat{y}})}{\sqrt{\sum (y_i - \bar{y})^2 \sum (\hat{y}_i - \bar{\hat{y}})^2}}$$

Spearman correlation coefficient was computed as:

$$\boldsymbol{\rho = 1 - \frac{6 \sum d_i^2}{n(n^2 - 1)}}$$

The coefficient of determination was calculated as:

$$R^2 = 1 - \frac{\sum (y_i - \hat{y}_i)^2}{\sum (y_i - \bar{y})^2}$$

Predictive squared correlation was calculated as:

$$Q^2 = 1 - \frac{PRESS}{\sum (y_i - \bar{y})^2}$$

Prediction errors were quantified using:

$$RMSE = \sqrt{\frac{1}{n} \sum (y_i - \hat{y}_i)^2}$$

and

$$MAE = \frac{1}{n} \sum | y_i - \hat{y}_i |$$

**where $y_i$and $\hat{y}_i$represent observed and predicted pIC<sub>50</sub> values, respectively.*

Supplementary Table S3

Statistical metrics

| Metric | Interpretation |
|---|---|
| **PCC** | Linear correlation between observed and predicted values |
| **Spearman** | Rank correlation |
| **$R^2$** | Goodness-of-fit |
| **$Q^2$** | Predictive ability |
| **RMSE** | Average prediction error |
| **MAE** | Mean absolute prediction error |

Supplementary Table S4

**Supplementary Table S4.**
Summary of the structural quality metrics of the predicted three-dimensional models for HSV glycoproteins generated using the SWISS-MODEL server

| Glycoprotein | Gene Bank Accession # | GMQE | QMEANDisCo Global |
|---|---|---|---|
| **gC** | AFH78104.1 | 0.23 | 0.46 ± 0.05 |
| **gE** | AYG87808.1 | 0.31 | 0.45 ± 0.11 |
| **gG** | AYG87875.1 | 0.08 | 0.095± 0.011 |
| **Gi** | AAW49017.1 | 0.14 | 0.37 ± 0.07 |
| **gJ** | AOY34336.1 | 0.06 | 0.22 ± 0.12 |
| **gK** | AAB60558.1 | 0.03 | 0.45 ± 0.12 |
| **gL** | AFH78105.1 | 0.58 | 0.70 ± 0.06 |
| **gM** | AOY34334.1 | 0.00 | 0.25 ± 0.12 |
| **gN** | AOY34292.1 | 0.26 | 0.64 ± 0.12 |
